# Ethanol Impairs Intestinal Barrier Function in Humans through Mitogen Activated Protein Kinase Signaling: A Combined *In Vivo* and *In Vitro* Approach

**DOI:** 10.1371/journal.pone.0107421

**Published:** 2014-09-16

**Authors:** Elhaseen Elamin, Ad Masclee, Freddy Troost, Harm-Jan Pieters, Daniel Keszthelyi, Katarina Aleksa, Jan Dekker, Daisy Jonkers

**Affiliations:** 1 Top Institute Food and Nutrition (TIFN), Wageningen, the Netherlands; 2 Division Gastroenterology-Hepatology, Department of Internal Medicine, Maastricht University Medical Center, Maastricht, the Netherlands; 3 Division of Clinical Pharmacology and Toxicology, Hospital for Sick Children, Toronto, Canada; 4 Host microbe interactomics, Department of Animal Sciences, Wageningen University, Wageningen, the Netherlands; Sezione di Gastroenterologia, Italy

## Abstract

**Background:**

Ethanol-induced gut barrier disruption is associated with several gastrointestinal and liver disorders.

**Aim:**

Since human data on effects of moderate ethanol consumption on intestinal barrier integrity and involved mechanisms are limited, the objectives of this study were to investigate effects of a single moderate ethanol dose on small and large intestinal permeability and to explore the role of mitogen activated protein kinase (MAPK) pathway as a primary signaling mechanism.

**Methods:**

Intestinal permeability was assessed in 12 healthy volunteers after intraduodenal administration of either placebo or 20 g ethanol in a randomised cross-over trial. Localization of the tight junction (TJ) and gene expression, phosphorylation of the MAPK isoforms p38, ERK and JNK as indicative of activation were analyzed in duodenal biopsies. The role of MAPK was further examined *in vitro* using Caco-2 monolayers.

**Results:**

Ethanol increased small and large intestinal permeability, paralleled by redistribution of ZO-1 and occludin, down-regulation of ZO-1 and up-regulation of myosin light chain kinase (MLCK) mRNA expression, and increased MAPK isoforms phosphorylation. In Caco-2 monolayers, ethanol increased permeability, induced redistribution of the junctional proteins and F-actin, and MAPK and MLCK activation, as indicated by phosphorylation of MAPK isoforms and myosin light chain (MLC), respectively, which could be reversed by pretreatment with either MAPK inhibitors or the anti-oxidant L-cysteine.

**Conclusions:**

Administration of moderate ethanol dosage can increase both small and colon permeability. Furthermore, the data indicate a pivotal role for MAPK and its crosstalk with MLCK in ethanol-induced intestinal barrier disruption.

**Trial Registration:**

ClinicalTrials.gov NCT00928733

## Introduction

Ethanol (ethyl alcohol) consumption is associated with several gastrointestinal (GI) and liver disorders, especially alcoholic liver disease (ALD) [1]. ALD is a progressive disease initiated by steatosis and inflammation, followed by liver fibrosis and cirrhosis [2]. Only 30% of chronic alcoholics eventually develop cirrhosis, indicating that additional factors are required [3]. Recent evidence points to a role for the gut-liver axis in the pathogenesis of ALD [4]. Ethanol is known to disrupt GI epithelial barrier integrity [5], resulting in translocation of potentially harmful bacteria and their products such as endotoxins [6] and peptidoglycans [7] into the portal circulation and consequently, liver injury. Dysfunction of GI mucosal barrier may result in increased susceptibility not only to infections [8] but also to development of ethanol-related GI cancers [9].

Human studies investigating effects of ethanol on intestinal barrier function have focused mainly on chronic heavy drinkers (>80 g/day) [10], demonstrating that ethanol increases small intestinal permeability [11]. Effects of moderate ethanol consumption on GI barrier function are less well known but are especially relevant since daily moderate alcohol consumption (daily intake of 1–2 alcoholic drinks or 12–24 g ethanol), is widespread [12]. Ethanol and its metabolites can reach the distal GI tract through the vascular space [13]. Therefore, ethanol and its metabolites may be injurious not only to the small but also to the large intestinal barrier. Intestinal barrier integrity is maintained by the tight junctions (TJs), a complex meshwork of transmembrane and cytoplasmic proteins including occludin, the claudins and the zona occludens family (ZO-1,2,3), linked to the cytoskeletal protein F-actin [14]. The TJs are supported by the adherens junctions proteins (AJs) E-cadherin and β-catenin, which are also required for TJ assembly [15]. Data obtained *in vitro* using intestinal epithelial cells (Caco-2 cell monolayers) indicate that ethanol disrupts TJs integrity via mechanisms involving oxidative stress [11], modulation of microtubules [16] and activation of the cell signaling pathway myosin light chain kinase (MLCK) [17]. The three isoforms of mitogen activated protein kinase (MAPK) isoforms including P38, extracellular signal-regulated kinase (ERK) and stress-activated protein kinase/C-Jun N-terminal kinase (SAPK/JNK) have been shown to modulate TJs integrity [18]. Furthermore, activation of MAPK has been reported to mediate intestinal epithelial barrier dysfunction [19]. Data on mechanims of ethanol-induced barrier dysfunction are mainly derived from *in vitro* studies [20]. In addition, insight into effects of moderate doses of ethanol on human intestinal barrier function and potential mechanisms involved is still lacking.

Our aims were to determine effects of a single moderate dose of ethanol, administered intraduodenally, on 1) small intestinal and colon permeability and 2) localization and expression of TJ in duodenal biopsies. In addition, the role of MAPK pathway as a primary signaling mechanism involved in ethanol-induced barrier disruption was investigated both *in vivo* in duodenal biopsies and *in vitro* in Caco-2 cell monolayers.

## Subjects and Methods

### Subjects and study design

The protocol for this trial and supporting CONSORT checklist are available as supporting information; see Checklist S1 and Protocol S1. The study was performed according to a randomized placebo-controlled crossover design, in 12 healthy subjects recruited between October 2010 and July 2011. Participants were healthy men from Caucasian ethnicity, between 18 and 45 years of age to avoid genome-related differences in ethanol metabolism [21]. Exclusion criteria included BMI>35 kg/m^2^, history of GI disorders, liver diseases i.e. hepatitis and cirrhosis, abdominal surgery, excessive alcohol consumption (>20 g/day) and smoking. The study flow diagram is shown in Figure S1. The study protocol was approved by the Ethics Committee of Maastricht University Medical Center (MUMC), conducted in accordance with the Declaration of Helsinki and registered at the Clinical Trial Registry (www.clinicaltrials.gov; NCT00928733). All participants provided written informed consent. An overview of the experimental procedures is given in Figure S1. The study consisted of two test days in a random order, with a washout period of one week. Participants arrived at the MUMC at 08:00 AM after an overnight fasting. An intravenous cannula was placed in an antecubital vein for blood sampling. Then, the tube was placed intraduodenally and optimal positioning was confirmed by X-ray.

Moderate ethanol intake is difficult to define. However, despite the complexity, Department of Agriculture and Department of Health and Human Services define moderate consumption of one standard drink a day for women and two drinks men [22]. A standard drink is generally considered to be 360 ml of beer, 150 ml of wine, or 45 ml of 80-proof distilled spirits. These drinks contain roughly the same amount of absolute alcohol-approximately 12–24 g [12]. Accordingly and via the intestinal tube, either 20 g ethanol in 100 ml water (5% v/v) or water (placebo) was perfused at a rate of 7 ml/min for 15 min. Earlier observations have indicated that ethanol can reach concentrations of 2–10% in small intestinal lumen after consumption of 50 ml ethanol in 20% solution [23]. Ethanol was introduced directly into the duodenum to overcome interindividual variations in its upper GI metabolism after oral consumption [24]. Thereafter, participants received an oral multi-sugar drink to assess intestinal permeability, consisting of 1 g lactulose (Centrafarm, Etten-Leur, the Netherlands), 1 g sucralose (Brenntag, Sittard, the Netherlands), 1 g erythritol (Danisco, Copenhagen, Denmark), 1 g sucrose (Van Gilse, Dinteloord, the Netherlands), and 0.5 g L-rhamnose (Danisco) dissolved in 150 ml tap water. At t = 30 min after start of placebo or ethanol perfusion, participants underwent a gastroduodenoscopy to obtain mucosal biopsies from the second part of the duodenum (forceps diameter: 2.8 mm). Tissue samples were frozen immediately in liquid nitrogen and stored at −80°C until further analyses. Blood was collected before and 15, 30, 45, 60, 90, 120, 150 and 180 min after onset of ethanol perfusion. Urine was collected in plastic containers before, and in three fractions after ethanol perfusion, i.e. 0–2 h, 2–5 h and 5–24 h. All samples were stored at −80°C within 4 hours after collection. For safety, participants were not allowed to leave the hospital unless their breath ethanol concentration was <0.05%, the legal limit in the Netherlands.

### Measurements

#### Liver function, blood ethanol and fatty acid ethyl esters (FAEEs) levels

Plasma alanine aminotransferase (ALT) and γ-glutamyltransferas (γGT) were determined before and 60 min after ethanol perfusion. Blood ethanol concentrations were determined by headspace gas chromatography aided with Flame Ionization Detection System with a detection limit of 10 mg/dL. Plasma concentrations were converted to blood alcohol concentration (BAC) using the conversion factor 0.809. Plasma levels of fatty acid ethyl esters (FAEEs) including ethyl oleate and ethyl palmitate were analyzed by gas chromatograph with a mass selective detector GC-MS QP-2010 PLUS equipped with GCMS solutions Software (Shimadzu, Maryland, U.S.A) [25].

#### Assessment of intestinal barrier function

Intestinal barrier function was assessed using a multi-sugar recovery test. Urinary sugar recovery was quantified by combined HPLC (Model PU-1980 pump, Jasco Benelux, Maarsen, the Netherlands) and mass spectrometry (Model LTQXL, Thermo Electron, Breda, the Netherlands) as described by van Wijck *et al*. [26]. Sucrose in 0–2 h urine, Lactulose/rhamnose (L/R) ratio in 0–5 h urine and sucralose/erythritol (S/E) ratios in the 5–24 h urine fraction were determined as indicators for gastroduodenal, small and large intestinal permeability, respectively.

#### Localization and gene expression of ZO-1 and occludin in duodenal mucosa

Immunofluorescent staining was performed as described previously [27], with minor modifications [27]. Tissue sections were examined under Leica TCS SPE confocal laser scanning microscope (Leica Micro systems GmbH, Mannheim, Germany), images were acquired at 512×512 pixel resolutions and z-stacks were obtained using Leica Application Suite Advanced Fluorescence software (Leica Microsystems GmbH). Images were processed and quantified as described by Fisher *et al*. [28], with minor modifications. Briefly, fluorescent staining of ZO-1 and occludin in the TJ region from uniform Z sections perpendicular to the apical cell surface of the epithelium were subjected to projection and passed through a Gaussian smoothing filter. Plot profiles of the staining intensity along the perpendicular lines were generated using Image J software [29]. Next, grey scale values of each image were calculated and normalized against a summed maximum intensity matrix (512×512×256) [28].

Gene expression of occludin, claudin 3, and claudin 4, myosin light chain kinase (MLCK) and ZO-1 in duodenal mucosal biopsies was evaluated by qPCR as described previously [30]. Primer sequences are given in the Table S1.

#### Assessment of MAPK isoforms phosphorylation in duodenal biopsies

Frozen tissue samples were homogenized in ice-cold PBS containing protease and phosphatase inhibitor cocktail (10 µl/ml PBS; Sigma-Aldrich). Concentrations of soluble proteins were quantified in tissue supernatants using the bicinchoninic acid assay (BCA; Bio-Rad). Lysates of Hela cells treated with Anisomycin were used as positive controls for phosphorylated P38 and JNK, whereas A431 cells treated with recombinant human epidermal growth factor were used for ERK1/2. Phosphorylated forms of P38, ERK1/2 and JNK in tissue and cell lysates were analysed using semi-quantitative sandwich ELISA according to manufacturer's instructions (Ray Biotech), and data are expressed as optical density (OD) at 450 nm.

#### Functional and structural assessment of epithelial barrier in Caco-2 cell monolayers

Colon adenocarcinoma cell line (Caco-2) from ATCC (Rockville, USA; passage 30–40) were maintained in Dulbecco's Modified Eagle Medium (DMEM; Lonza Benelux BV, Breda, the Netherlands) and barrier permeability was determined using Caco-2 monolayers grown on collagen-coated permeable polycarbonate Transwell filters (Costar, Cambridge, MA, USA) as described previously [16]. Briefly, monolayers were exposed to 40 mM ethanol for 3 h, either alone or after pretreatment for 1 h with 100 µM SB2035809 (P38 inhibitor; Cell Signaling Technology, MA, USA), PD98059 (ERK 1/2 inhibitor; Cell Signaling Technology, MA, USA) or SP600125 (JNK inhibitor; Selleckchem, TX, USA) in medium. Thereafter, barrier function was assessed by measuring transepithelial electrical resistance (TEER) and apical to basolateral permeation of the fluorescent marker fluorescein isothiocyanate-labeled dextran 4 KDa (1 mg/ml FITC-D4; Sigma-Aldrich) using an EVOM Epithelial Voltohmmeter (World Precision Instruments, Berlin, Germany) and spectrophotometer at an excitation and emission wavelengths of 498 nm and 540 nm, respectively, and permeability was quantified as percentage of TEER and FITC-D4 permeating to the basal compartment.

Following subjection to the aforementioned treatments, Caco-2 monolayers were fixed on the inserts for 10 min with 4% (w/v) paraformaldehyde and permeabilized with 0.1% (v/v) Triton X-100 in PBS at RT for 40 min. Next, monolayers were processed for immunofluorescence staining of the TJ proteins ZO-1 and occludin, AJ proteins E-cadherin and β-catenin, and F-actin as we described previously [16].

#### Determination of MAPK isoforms and MLC protein phosphorylation in Caco-2 cells

Phosphorylation of P38, ERK1/2 and JNK, and MLC as indicative of MAPK and MLCK activation was determined using cell-based ELISA kits (Ray Biotech Inc, Norcross, GA, USA.) according to manufacturer's instructions. Briefly, Caco-2 cells (20×10^3^) were seeded in 96 well-plates (Corning BV) and incubated overnight at 37°C, 5% CO_2_. Monolayers were treated as described earlier. In some experiments, monolayers were also treated with 50 µM of MLCK inhibitor 1-(5-iodonaphthalene-1-sulfonyl)-1H-hexahydro-1, 4-diazepine (ML-7). Next, monolayers were fixed and blocked and were incubated with rabbit anti: total and phosphorylated P38 (p-P38), ERK1/2 (p-ERK1/2) and JNK (p-JNK, 1∶100 dilution in the blocking solution; Ray Biotech), and phosphorylated MLC (p-MLC1∶100 dilution in the blocking solution; Cell Signaling Technology), followed by HRP-conjugated mouse anti-rabbit IgG (Dako, Glostrup, Denmark). Finally, 3, 3′, 5, 5′-Tetramethylbenzidine (TMB) was added, followed by stop solution and optical density was read at 450 nm by SpectraMax M2 spectrophotometer (Molecular Devices).

#### Power calculation and statistical analyses

Primary outcome was intestinal permeability. Based on study by Troost *et al*. [31] comparing small intestinal permeability before and after intake of indomethacin in15 healthy volunteers, lactulose/rhamnose (L/R) ratio increased from 0.013±0.009 to 0.031±0.020 after indomethacin treatment. In the above mentioned study, the standard deviation (SD) in the indomethacin-treated group was 0.02 and the mean difference was 0.018. In our study, each subject matched to one control and to detect comperable differences in small intestinal permeability using SD of 0.02, 12 healthy volunteers were needed to be able to reject the null hypothesis that the population means of the experimental and control groups are equal using the probability power β = 0.8 and the type I error probability α = 0.05. This sample size calculation was performed with PS-Power and Sample Size Program version 3.0. Statistical analyses were performed using GraphPad Prism software (version 5, Windows, San Diego, CA, USA). Data were tested for normality by the Kolmogorov-Smirnoff test. Normally distributed data were analyzed by independent Student's t test. Wilcoxon's and Mann-Whitney U tests were used for abnormally distributed data. *In vitro* data, each conducted in triplicate, are presented as means ± SD of at least three experiments. Data were analysed using one-way analysis of variance (ANOVA) followed by Bonferroni multiple comparisons and P-values adjustment (Bonferroni correction), and *P*<0.05 was considered statistically significant.

## Results

### Subjects

Twelve male healthy volunteers [age 30.8±3.1 years; BMI 23.0±0.07 kg/m^2^] participated in this study. None of the participants experienced any GI discomfort during the test days. No side effects occurred and no changes in plasma ALT and γGT levels after ethanol administration were observed (data not shown).

### Ethanol and FAEE

**Figure 1:**
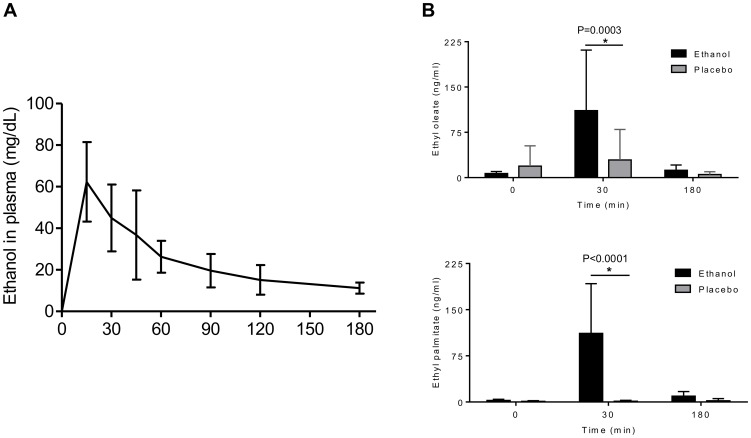
Effect of placebo or single dose of 20 g of ethanol administration on blood ethanol and FAEE levels. [**A**] Ethanol was analyzed in plasma of the individual volunteers after ethanol administration at the following time points: 0, 15, 30, 45, 60, 90, 120, and 180 min, and presented as mean ± SD. [**B**] Ethyl oleate and Ethyl palmitate were determined in plasma at 0, 30, and 180 min, and presented as mean ± SD (n = 12).

Blood samples collected prior to the intervention were negative for ethanol (i.e. <10 mg/dl plasma) and FAEEs. Following intraduodenal ethanol, plasma ethanol concentration peaked at 15 min (62 mg/dl) and gradually declined to 11 mg/dl at 180 min (Figure 1A). Mean plasma concentrations of the FAEEs ethyl oleate and ethyl palmitate were significantly increased 30 min after ethanol administration and decreased towards basal levels at 180 min (P = 0.0003 and P<0.0001, respectively; Figure 1B).

### Gastroduodenal, small and large intestinal permeability

**Figure 2:**
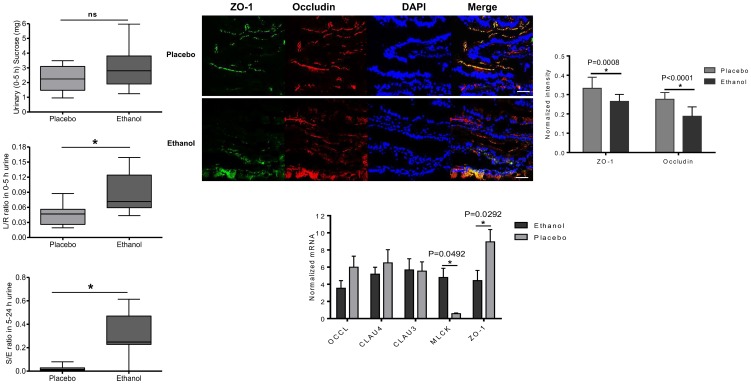
Effects of placebo and single dose of 20 g of ethanol on intestinal permeability, TJs protein and gene expression in the duodenum. [**A**] Effects on gastroduodenal permeability (sucrose, 0–5 h urinary recovery). Data presented as boxplots displaying minimum, maximum, and the 25th, 50th and 75th percentiles (*n* = 12); *P*>0.05 vs. placebo. [**B**] Effects on small intestinal permeability (lactulose/rhamnose; L/R ratio, 0–5 h urine). Data presented as boxplots displaying minimum, maximum, and the 25th, 50th and 75th percentiles (*n* = 12); *P<0.01 vs. placebo. [**C**] Effects on large intestinal permeability (sucralose/erythritol; S/E ratio, 5–24 h urine). Data presented as boxplots displaying minimum, maximum, and the 25th, 50th and 75th percentiles (n = 11); *P<0.01 vs. placebo. [**D**] Representative images of immune-localization of ZO-1 (green), occludin (red), and nuclei (blue) at a 400x magnification are shown. Scale bar represents 10 µm. [**E**] Normalized intensity of immunofluorescent-labelled ZO-1 and occludin in duodenal mucosa. Z-stack images were at 40× objective power. Data are mean ± SD (n = 12). *P<0.05 for ZO-1, ^#^ *P*<0.05 for occludin. [**F**] Normalized mRNAs expression of occludin (occl), claudin 4 (clau4), and claudin 3 (clau3), MLCK and ZO-1 in duodenal biopsy specimens determined by qPCR. Each bar represents the mean± SD; *P<0.05 comparing ethanol with placebo.

There was no significant difference in mean urinary sucrose recovery between placebo (2.309±0.85) and ethanol (3.087±1.51), (P = 0.1349; Figure 2A). However, the L/R ratio, representing small intestinal permeability, was significantly higher after ethanol (0.089±0.04) compared to placebo (0.047±0.02), (P = 0.0076; Figure 2B). Furthermore, ethanol significantly increased the S/E ratio in 5–24 h urine, as indicator for large intestinal permeability, versus placebo (0.317±0.18 vs. 0.026±0.03, respectively), (P = 0.0007; Figure 2C).

### Localization and expression of TJ proteins in duodenal mucosa

After placebo administration, ZO-1 and occludin showed strong staining in duodenal biopsies at the apical part of epithelial cells, being continuous without disruption and showed colocalization along the villous epithelium. In contrast, after ethanol, ZO-1 and occludin showed less immune reactivity, disruption and complete absence of colocalization at the apical membrane compared with placebo (Figure 2D). These features were confirmed by measurement of normalized image intensity of immunolabeled ZO-1 and occludin in duodenal biopsies. The normalized intensity of both ZO-1 and occludin decreased significantly after ethanol versus placebo (P = 0.0008 and P<0.0001, respectively; Figure 2E). In addition, ethanol significantly down-regulated ZO-1 and up-regulated MLCK mRNA expression compared to placebo (P = 0.092 and P = 0.0492, respectively; Figure 2F). No differences were found in gene expression of occludin, claudin 3 and claudin 4 (P>0.05; Figure 2F).

### Phosphorylation of MAPK isoforms in duodenal mucosal biopsies

**Figure 3:**
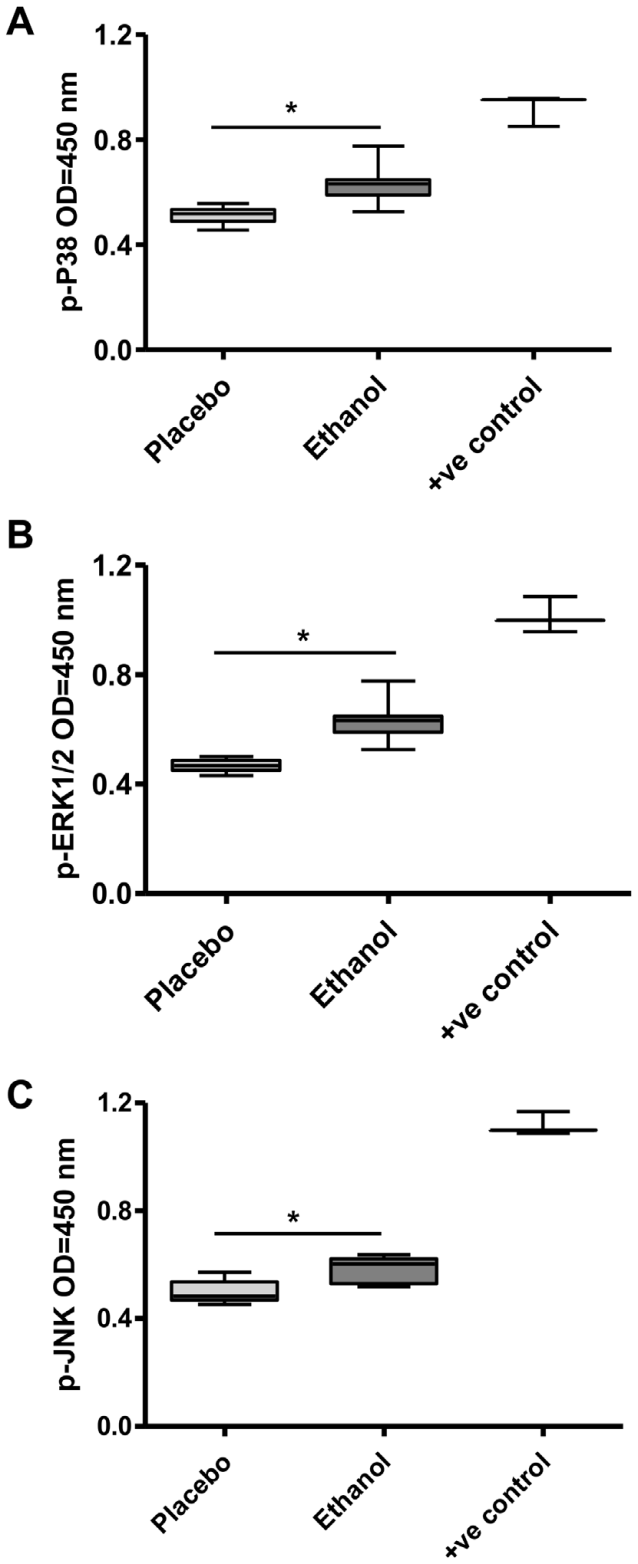
Effect of placebo and single dose of 20 g of ethanol on phosphorylation of MAPK isoforms in intestinal epithelium. Mucosal protein was isolated from tissue lysates, and the levels of phosphorylated MAPK isoforms were determined by ELISA using anti-phosphospecific antibodies against p-P38 [**A**], p-ERK1/2 [**B**], and [**C**] and p-JNK. Lysates of Hela cells treated with Anisomycin were used as positive control (+ve control) for phosphorylated P38 and JNK, whereas A431 cells treated with recombinant human epidermal growth factor were used for phosphorylated ERK1/2. (n = 12 separate sample preparations; *P<0.05 vs. placebo).

The phosphorylation of MAPK isoforms in duodenal mucosal biopsies after ethanol and placebo administration was checked quantitatively using ELISA. Results showed that ethanol significantly increased the level of phosphorylated P38 (p-P38; P<0.0001), ERK1/2 (p-ERK; P<0.0001) and JNK (p-JNK; P = 0.0001) compared to the placebo (Figure 3A, B and C, respectively).

### Phosphorylation of MAPK isoforms in Caco-2 cells

**Figure 4:**
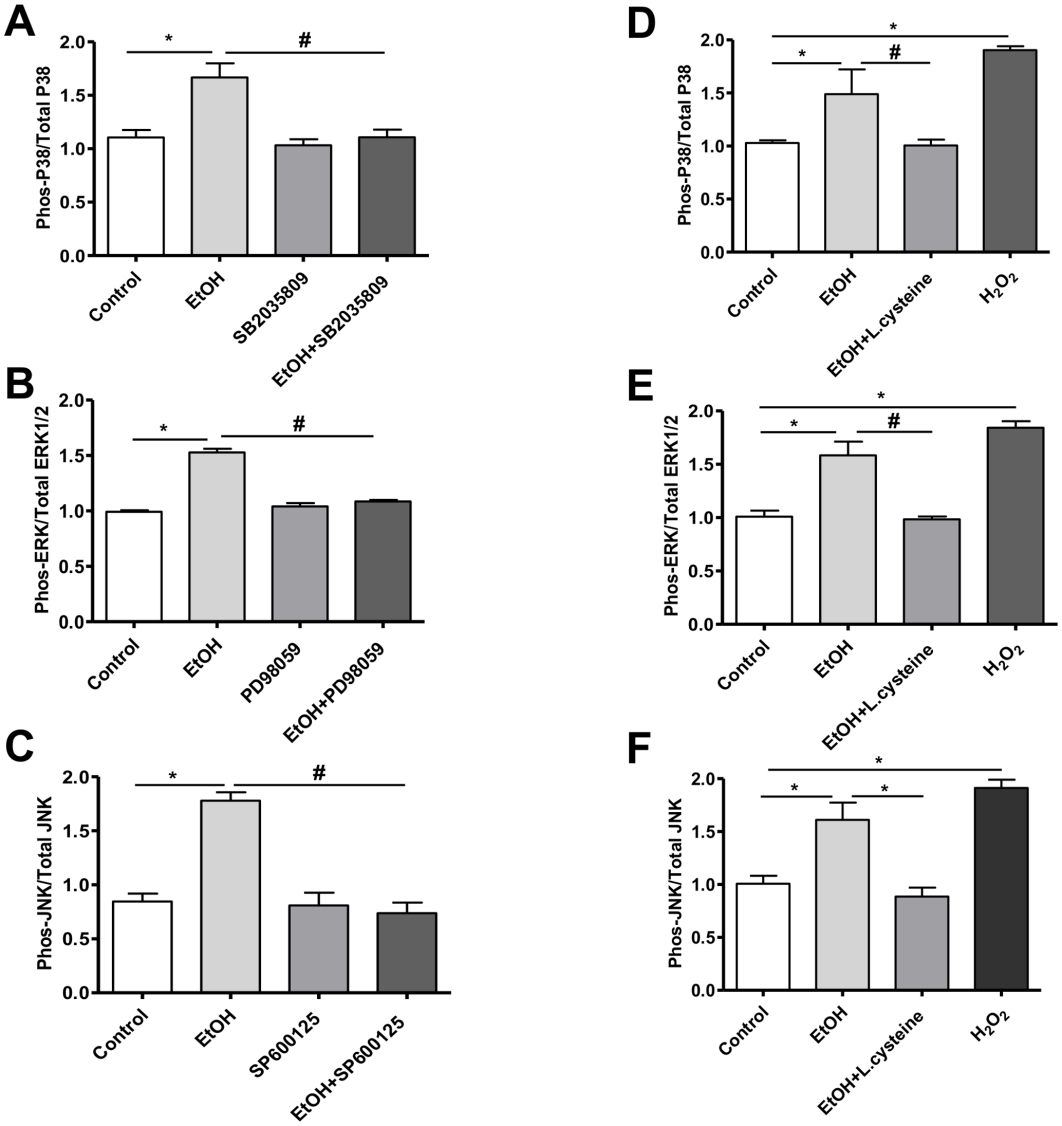
Effect of ethanol on phosphorylation of MAPK isoforms in Caco-2 cells. [**A**] Phosphorylation of P38, [**B**] ERK1/2, and [**C**] JNK was determined by cell-based ELISA and expressed as the ratio of the phosphorylated to the total form of each protein. n = 3 and *P<0.0001 vs. control and ^#^ *P*<0.0001 vs. ethanol in the presence of SB2035809, PD98055, or SP600125, respectively. Effect of L-cysteine on ethanol-induced phosphorylation of P38 [**D**], ERK1/2 [**E**], and JNK [**F**], determined by cell-based ELISA, and expressed as the ratio of the phosphorylated to the total amount of each protein. H_2_O_2_ used as positive control. n = 3 and *P<0.0001 vs. control and ^#^ *P*<0.0001 vs. ethanol after pretreatment with L-cysteine.

Next, we used Caco-2 cell monolayers to further elucidate the role of MAPK activation in ethanol-induced barrier disruption *in vitro* by assessment of their protein phosphorylation. Compared with control, 40 mM ethanol significantly induced activation of MAPK in Caco-2 cells, indicated by increased p-P38/total P38 (P = 0.0168; Figure 4A), p-ERK1/2/total ERK1/2 (P = 0.0002; Figure 4B), and p-JNK/total JNK ratio (P = 0.0034; Figure 4C). Activation of MAPK was completely prevented by pretreatment with the P38 kinase inhibitor SB2035809 (P = 0.0187; Figure 4A), ERK1/2 inhibitor PD98059 (P = 0.0003; Figure 4B) and JNK inhibitor SP600125 (P = 0.0022; Figure 4C). To determine whether ethanol exposure causes MAPK activation via oxidative stress, Caco-2 cells were exposed to ethanol with or without L-cysteine, the rate limiting substrate for glutathione synthesis. Pretreatment with L-cysteine (100 µM) significantly attenuated ethanol-induced activation of P38 (P = 0.0075; Figure 4D), ERK1/2 (P<0.0001; Figure 4E), and JNK ((P = 0.0002; Figure 4F).

### MAPK activation in ethanol-induced barrier disruption in Caco-2 monolayers

**Figure 5:**
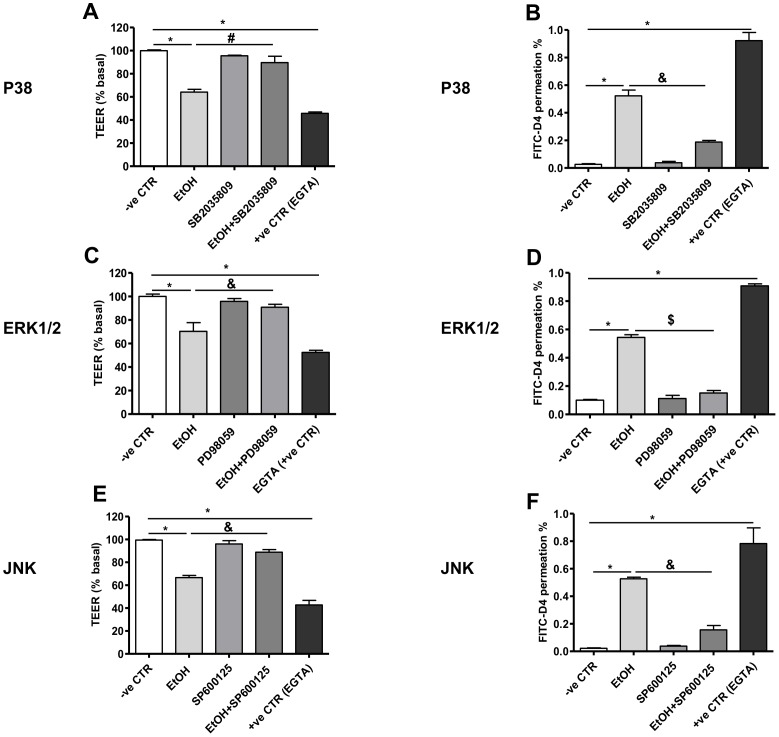
Effects of MAPK inhibition on ethanol-induced barrier dysfunction in Caco-2 monolayers. Confluent human intestinal epithelial cells (Caco-2) grown on inserts were assessed for trans-epithelial electrical resistance (TEER) and permeability to FITC-D4 after 3 h after treatment with P38 inhibitor [**A**] and [**B**], ERK1/2 inhibitor [**C**] and [**D**] and **JNK** inhibitor [**E**] and [**F**], respectively**.** Permeability is expressed as percentage of basal TEER and FITC-D4 permeation from apical to basal chamber. Some cells were treated only with medium or ethylene glycol tetra acetic acid (EGTA) as negative (-ve CTR) and positive control (+ve CTR), respectively. In addition, some monolayers were pretreated for 1 h with 100 µM of the P38-specific kinase inhibitor SB2035809, ERK1/2 PD98055 and JNK SP600125. Data are means ± SD of triplicate wells from three separate experiments. *P<0.0001 vs. control, ^#^ *P*<0.001, ^&^ *P*<0.0001 and ^$^ *P*<0.05 vs. ethanol after pretreatment with each MAPK inhibitor.

**Figure 6:**
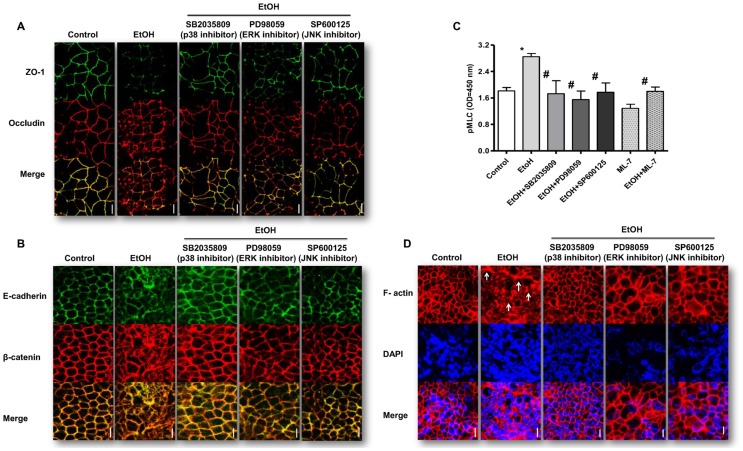
Effects of inhibition of MAPK isoforms on ethanol-induced redistribution of TJ and AJ proteins; activation of MLCK and reorganization of F-actin cytoskeleton in Caco-2 monolayers. Monolayers subjected to aforementioned treatments were washed, fixed, permeabilized, and double-stained for ZO-1, occludin and E-cadherin, and ß-catenin. [**A**] Representative images of immunostaining of ZO-1 (*green*) and occludin (*red*) of three independent experiments. Scale bar  = 10 µm. [**B**] Representative images of immunostaining of E-cadherin (*green*) and β-catenin (*red*). Scale bar  = 10 µm. [**C**] Effects of MAPK inhibition on ethanol (EtOH)-induced myosin light chain (MLC) phosphorylation. Caco-2 cell monolayers were incubated with 40 mM ethanol (EtOH) for 3 h with or without MAPK inhibitors (as in Figure 6) or MLCK inhibitor (ML-7) pretreatment for 1 h, and analyzed by cell-based ELISA, using phosphospecific MLC antibodies (n = 3). Each bar represents the mean ± SD; *P<0.0001 vs. control and ^#^ *P*<0.0001 vs. ethanol. [**D**] Effects of MAPK inhibition on ethanol (EtOH)-induced F-actin reorganization. Caco-2 cell monolayers incubated with 40 mM ethanol for 3 h with or without MAPK inhibitors pretreatment for 1 h were fixed and stained for F-actin with Texas red-X phalloidin Scale bar  = 10 µm.

To evaluate the role of MAPK in effects of ethanol on intestinal barrier function, pharmacological inhibitors of its isoforms were used. Caco-2 monolayers exposed to ethanol showed a significant decrease in TEER (P<0.0001, P<0.0001, P<0.0001; Figure 5A, C and E, respectively) and increased FITC-D4 permeation (P<0.0001, P<0.0001, P<0.0001; Figure 5B, D, and F, respectively) compared to controls. In contrast, monolayers pretreated with the MAPK isoforms inhibitors SB2035809, PD98059 and SP600125 showed no drop but significant increase in TEER (P<0.0001; P = 0.0002, P<0.0001; Figure 5A, C and E, respectively) and significant decrease in FITC-D4 permeation (P<0.0001, P<0.0001, P<0.0001; Figure 5B, D, and F, respectively) in response to ethanol treatment. Compared to controls, treatment of Caco-2 monolayers with 40 mM ethanol induced delocalization and internalization of ZO-1 and occludin (Figure 6A), E-cadherin and β-catenin from apical membranes to intracellular compartments (Figure 6B). However, inhibition of MAPK isoforms with SB2035809, PD98059 and SP600125 prevented ethanol-induced changes in localization of the TJ (Figure 6A), and AJ (Figure 6B). Ethanol-induced intestinal barrier dysfunction may involve MLCK activation, as indicated by MLC phosphorylation [17]. As shown in Figure 6C, ethanol significantly increased MLC phosphorylation in Caco-2 monolayers compared to controls (P<0.0001). However, inhibition of MAPK with SB2035809, PD98059 and SP600125 significantly attenuated ethanol-induced induced MLC phosphorylation (P<0.0001; P<0.0001, P<0.000, respectively; Figure 6C), which was comparable to effects of the specific MLCK inhibitor 1-(5-iodonaphthalene-1-sulfonyl)-1H-hexahydro-1, 4-diazepine (ML-7) [17]. Since MLCK activation induces contraction of the peri-junctional actomyosin ring resulting in loss of TJ integrity [32], our experiments were extended further to elucidate effects of MAPK inhibition on ethanol-induced actin cytoskeleton rearrangement by immunofluorescence microscopy. As shown in figure 6D, in control condition, basal F-actin is uniformly distributed and organized in ring-like structures around the cellular periphery. However, in ethanol-treated monolayers, F-actin appears disorganized with thickened stress fibers (Figure 6D, arrowhead). Inhibition of MAPK isoforms by SB2035809, PD98059 and SP600125 attenuated ethanol-induced remodeling of actin cytoskeleton (Figure 6D).

## Discussion

This study demonstrates that administration of a single moderate ethanol dose increases both small and large intestinal permeability in healthy volunteers.The impairment of intestinal barrier function was paralleled by redistribution of ZO-1 and occludin, down-regulation of ZO-1 and up-regulation of MLCK gene expression, and activation of MAPK isoforms (i.e.p38, ERK1/2 and JNK) in duodenal mucosa. *In vitro* data confirmed that ethanol induces MAPK isoforms phosphorylation, together with changes in permeability, redistribution of ZO-1 and occludin, disruption of F-actin ring and a significant increase in phosphorylation of MLC. These changes were reversed by the anti-oxidant L-cysteine and by MAPK inhibitors. Previous studies reporting on effects of ethanol intestinal barrier function have mainly been performed in alcoholics [20]. We are the first to report on effects of a moderate dose of ethanol on intestinal epithelial barrier combining data at functional level with data of epithelial TJs at transcriptional and posttranscriptional level focusing on potential mechanisms.

At functional level, our results are in line with those of Robinson *et al*. [33] showing that a single moderate dose of ethanol increases excretion of urinary PEG 400, pointing to increased small intestinal permeability. We did not observe any effect on gastroduodenal permeability in the present study, in contrast to previous reports [5], [34]. This is not surprising given the fact that ethanol was administered via a tube directly into the distal duodenum, bypassing the stomach and proximal duodenum.

Interestingly, we found not only increase in small intestinal permeability but also in colon. While an increase in colon permeability by ethanol has been reported in animal studies, so far no data are available with respect to the human condition. In rats, ethanol (3–4.5 g/kg) gavage for 12 days was found to increase colon permeability. This could be prevented by antibiotics and also by mast cell membrane stabilizers, pointing to involvement of colon microbiota and mast cell activation [35].

After ingestion, ethanol can reach colon mucosa by diffusion from blood [13]. Intestinal bacteria exhibit alcohol dehydrogenase (ALD) and to a lesser extent aldehyde dehydrogenase (ALDH) activity [36] with a lower capacity to metabolize acetaldehyde [37]. Together with less active ALDH in colon mucosa [38], this will result in intracolonic accumulation of acetaldehyde. Increased intracolonic accumulation of acetaldehyde is considered an important factor contributing to colorectal carcinogenesis [39] and hepatotoxicity [40].

Little is known about the duration of the increments in small and large intestinal permeability by ethanol. Robinson *et al*. [33] found the increase in small intestinal permeability after a single ethanol dose to be transient. This does not exclude that prolonged changes may occur after repeated, chronic ethanol exposure. Ethanol consumption has been associated with elevation of plasma endotoxins [41] and increased drug absorption [42], possibly through changes in intestinal barrier function. Recent data indicate that moderate red wine consumption (i.e. 20 g ethanol per day) by patients with inflammatory bowel diseases (IBD) in remission, in whom intestinal permeability already is increased, even further increases both small and large intestinal permeability [34].

We found that ethanol-induced increase in small intestinal permeability was associated with disruption of the key TJ proteins ZO-1 and occludin, and modulated mRNA expression of ZO-1 and MLCK in duodenal biopsies. Timing of biopsy sampling, i.e. 30 min after ethanol perfusion, was based on plasma ethanol levels. Mechanistic studies in humans on effects of moderate ethanol intake are lacking. However, in chronic alcoholics, Tang *et al*. [43] have reported increase in microRNA 212 expression in colon mucosa with subsequent post-transcriptional suppression of ZO-1 synthesis.

*In vitro*, down-regulation of ZO-1 and activation of MLCK has been shown to mediate ethanol-induced TJ disruption and actin cytoskeleton rearrangement, and consequently, increased intestinal epithelial permeability [17]. Other cell signaling pathways including MAPK have also been proposed as mechanism for ethanol-induced barrier dysfunction [44]. MAPK isoforms including p38, extracellular signal-regulated kinase (ERK) and stress-activated protein kinase/C-Jun N-terminal kinase (SAPK/JNK) represent a converging point for many signaling pathways and have been linked to broad spectrum of cellular responses to extracellular stimuli such as growth factors and stress [45]. In the present study, we observed that ethanol induced phosphorylation of p38, ERK1/2 and JNK in duodenal mucosa, indicating their activation. These observations led us to further explore their role in Caco-2 cell monolayers. Our *in vitro* results confirmed that ethanol exposure, at a concentration that can be achieved in blood after moderate consumption, can induce MAPK activation akin to H_2_O_2_, a potent oxidative stress inducer. In addition, ethanol increased permeability and induced abnormal distribution of TJ proteins and AJ proteins, which could be attenuated by MAPK inhibition. Such effects were not found after pretreatment with the anti-oxidant L-cysteine, indicating that ethanol activates MAPK, at least in part, through an oxidative stress-dependent mechanism. This finding is in line with reports by others and by our group, identifying oxidative stress as a key factor in the mechanisms by which ethanol and its metabolites disrupt intestinal barrier function [46], [47]. Furthermore, oxidative stress has been found to activate MAPK in intestinal epithelial cells [48]. Therefore, our findings have relevance for ethanol-induced gut leakiness and indicate that the deleterious effects on barrier integrity can be reduced with antioxidants.

The inability of ethanol to induce changes in Caco-2 monolayers after pretreatment with MAPK inhibitors suggests that ethanol requires MAPK activation to disrupt TJ, highlighting a pivotal role for MAPK in ethanol-induced barrier disruption. Previously, it has been found that p38-dependent MLCK activation is required for burn-induced intestinal permeability [49]. Our study provided evidences that ethanol leads to MAPK-dependent MLC phosphorylation, F-actin rearrangement and stress fiber formation in Caco-2 cells. Disruption of actin cytoskeleton and interaction with TJ and AJ have been implicated in ethanol and acetaldehyde-induced loss of TJ integrity in Caco-2 cell monolayers [50]. Therefore, our *in vitro* data support the *in vivo* observations that ethanol increases intestinal permeability in association with up-regulation of MLCK gene expression and activation of MAPK in duodenal mucosa.

Ethanol and FAEEs including ethyl oleate and ethyl palmitate were measured in plasma, showing that ethanol can reach high levels within 30 min after administration accompanied by increased concentrations of the FAEEs ethyl oleate and ethyl palmitate. Acetaldehyde concentrations have not been measured due to its volatile character.

Duodenal biopsies obtained at the same time point (i.e. 30 min post-administration) have shown major changes on TJ at both posttranscriptional and transcriptional levels. Prior *in vitro* data have shown that ethanol [46], acetaldehyde [51] and FAEEs [47] can increase paracellular permeability in Caco-2 monolayers. Since intestinal mucosa is considered a major site for FAEE synthesis [52], together with a shift in ethanol metabolism into nonoxidative pathways resulting from low ALD activity in chronic alcoholics additive or even synergistic deleterious effects of ethanol and its oxidative and nonoxidative metabolites are expected to occur *in vivo* [53].

Limitations of our study include the lack of assessment of TJ integrity at the colon mucosal level. In addition, being a proof of concept study, ethanol was administered directly into the duodenum to overcome interindividual variations in its upper GI metabolism. Our data allow future studies to include more physiological approaches with a major focus on the colon.

In conclusion, a moderate dose of ethanol increases small and large intestinal permeability. Oxidative stress-mediated activation of MAPK is involved in ethanol-induced TJ and AJ disruption, actin cytoskeleton reorganization and consequently, intestinal epithelial barrier dysfunction. Our findings have potential implications for pathogenesis of alcoholic-related liver and GI disorders. A better understanding of molecular mechanisms involved in ethanol-induced gut leakiness may provide clues for development of preventive strategies.

## Supporting Information

Figure S1 **Flow diagram showing the process of enrolment, allocation, follow up and analysis of the study.**
(TIF)

Figure S2 **Timeline of the test day.** B, Blood sample; EtOH, Ethanol; G, Gastroduodenoscopy; S, Oral intake of sugars; Pl, Placebo.
(TIF)

Table S1 **Primer Sequences for RT-PCR.**
(TIF)

Checklist S1 **CONSORT checklist.**
(DOC)

Protocol S1 (DOC)
