# Supplementary figures and images for: Ethanol Impairs Intestinal Barrier Function in Humans through Mitogen Activated Protein Kinase Signaling: A Combined In Vivo and In Vitro Approach
Source: PLoS One. 2014 Sep 16;9(9):e107421. doi: 10.1371/journal.pone.0107421 (PMC4165763; doi:10.1371/journal.pone.0107421)

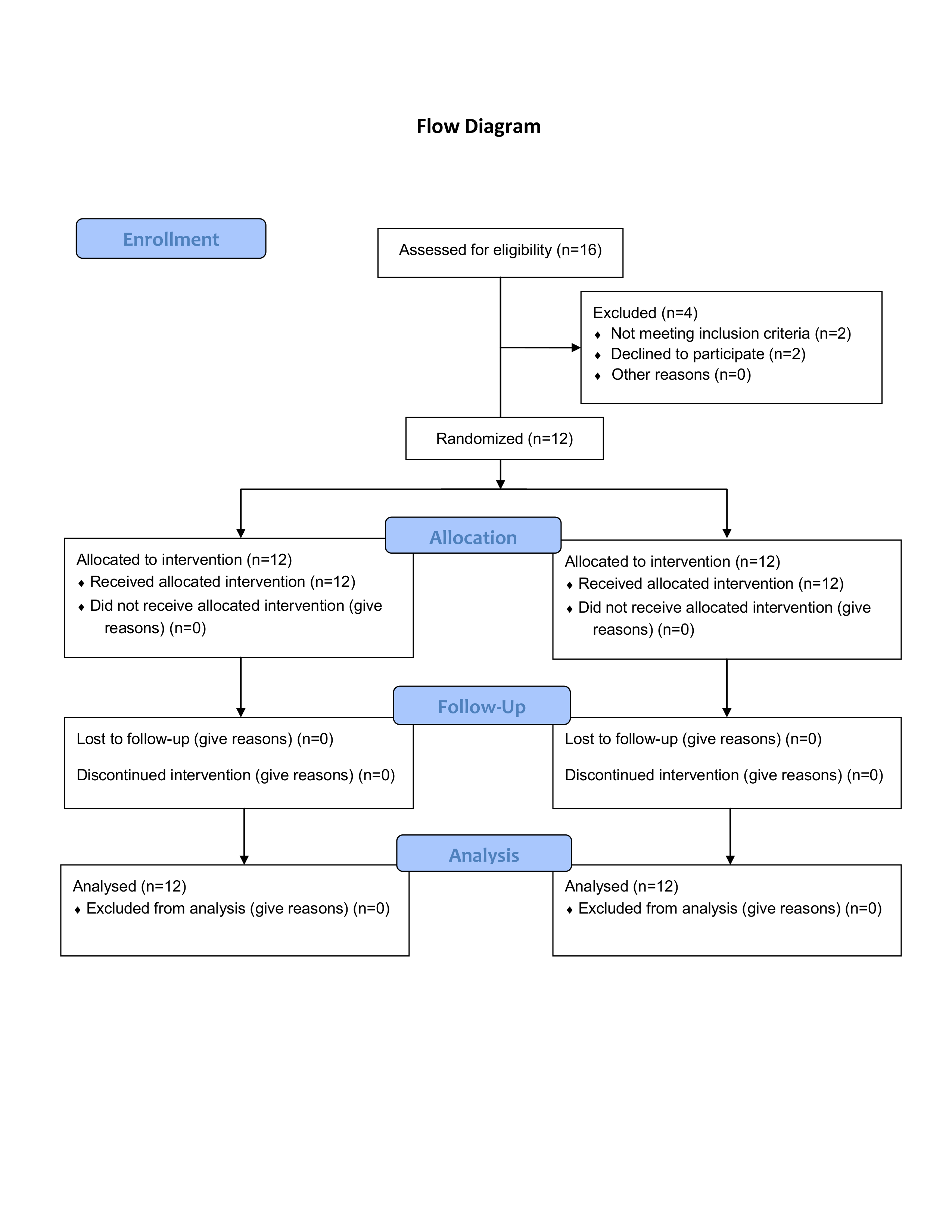

Supplement: Figure S1 — Flow diagram showing the process of enrolment, allocation, follow up and analysis of the study. (TIF) [file pone.0107421.s001.tif]

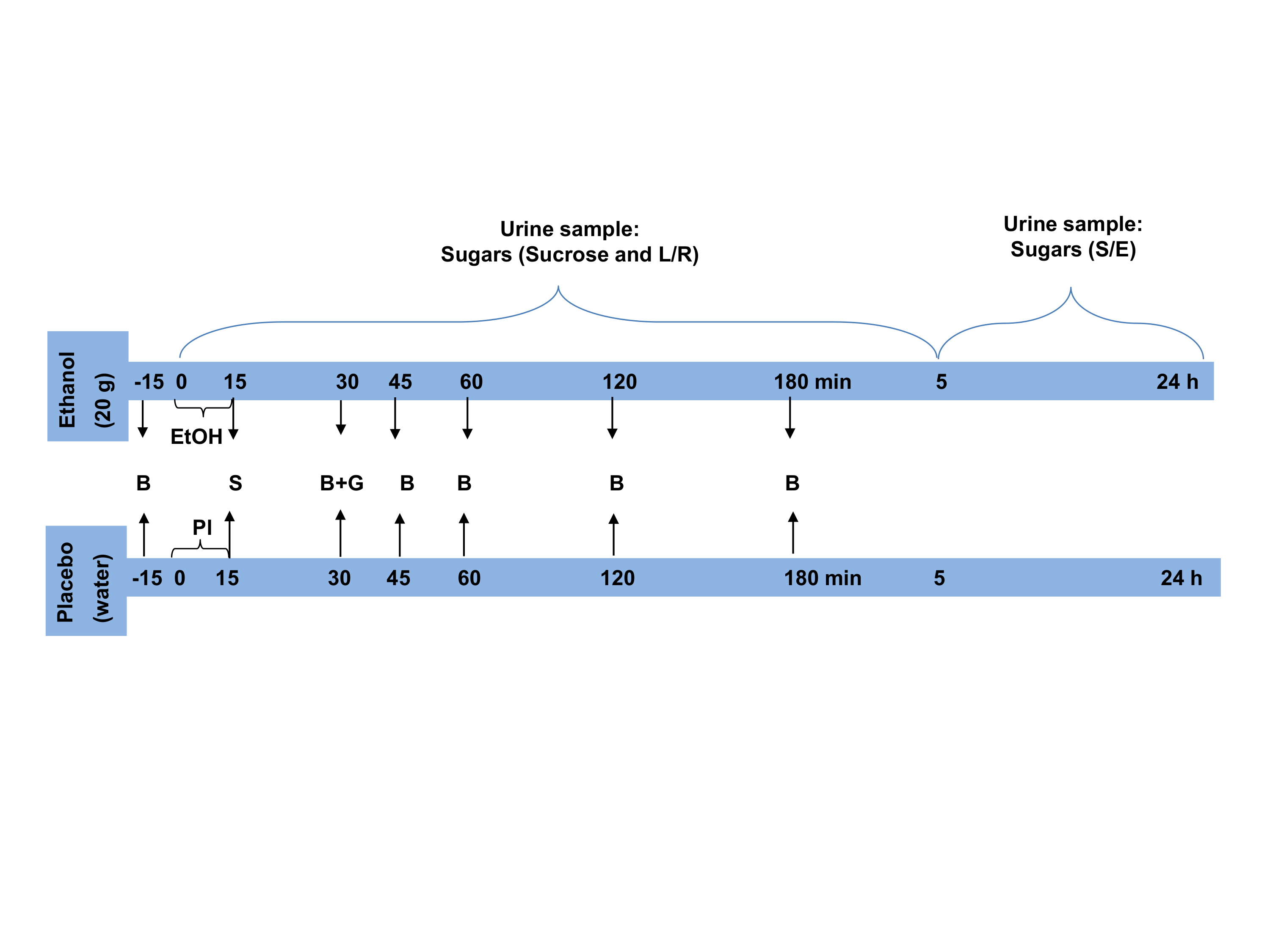

Supplement: Figure S2 — Timeline of the test day. B, Blood sample; EtOH, Ethanol; G, Gastroduodenoscopy; S, Oral intake of sugars; Pl, Placebo. (TIF) [file pone.0107421.s002.tif]

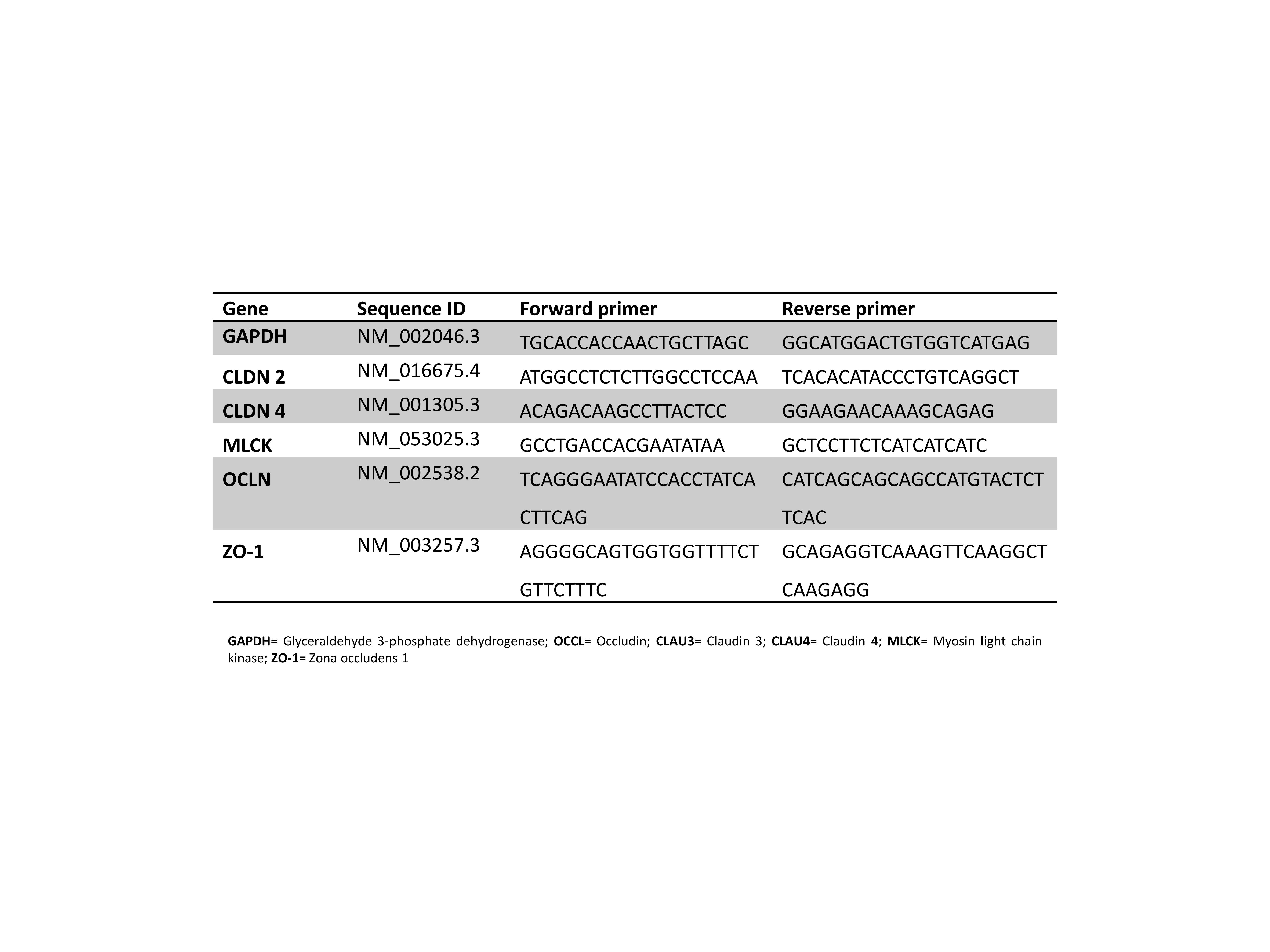

Supplement: Table S1 — Primer Sequences for RT-PCR. (TIF) [file pone.0107421.s003.tif]
