# Supplementary material for: Ethanol Impairs Intestinal Barrier Function in Humans through Mitogen Activated Protein Kinase Signaling: A Combined In Vivo and In Vitro Approach
Source: PLoS One. 2014 Sep 16;9(9):e107421. doi: 10.1371/journal.pone.0107421 (PMC4165763; doi:10.1371/journal.pone.0107421)
Supplement: Protocol S1 — (DOC) [file pone.0107421.s005.doc]

**Study proposal:**

**The effect of ethanol on intestinal permeability and integrity in**

**healthy individuals**

Department of Internal Medicine

Division of Gastroenterology and Hepatology

Maastricht University Medical Centre

PO Box 5800

6202 AZ Maastricht

The Netherlands

Protocol title

Short title: Ethanol and intestinal permeability

Coordinating investigator: Prof. Dr. A. Masclee

Division of Gastroenterology-Hepatology

Dept. of Internal Medicine

Maastricht University Medical Centre

043-3875021

Principal investigators: Prof. Dr. A. Masclee

Division of Gastroenterology-Hepatology

Dept. of Internal Medicine

Maastricht University Medical Centre

043-3875021

Dr. D. Jonkers

Division of Gastroenterology-Hepatology

Dept. of Internal Medicine

Maastricht University Medical Centre

043-3884266

*Independent physician: Dr. Y. Keulemans, MD (nog toestemming vragen)*

*Division of Gastroenterology-Hepatology*

*Dept. of Internal Medicine*

*Maastricht University Medical Centre*

*043-3875021*

Project coordinator TIFN: Dr. J. Dekker

Top Institute Food and Nutrition

Wageningen

0317-486173

Project team members: E. Elamin, MD, MSc

Division of Gastroenterology-Hepatology

Dept. of Internal Medicine

Maastricht University Medical Centre

043-3882983

Dr. F. Troost

Division of Gastroenterology-Hepatology

Dept. of Internal Medicine

Maastricht University Medical Centre

043-3884296

D. Keszthelyi, MD

Division of Gastroenterology-Hepatology

Dept. of Internal Medicine

Maastricht University Medical Centre

043-3881982

A. Kodde

Division of Gastroenterology-Hepatology

Dept. of Internal Medicine

Maastricht University Medical Centre

043-3884295

K. van Wijck, MD

Dept. of Surgery

Maastricht University Medical Centre

Dr. K. Lenaerts

Dept. of Surgery

Maastricht University Medical Centre

Prof. Dr. C. Dejong

Dept. of Surgery

Maastricht University Medical Centre

M. Hadfoune

Dept. of Surgery

Maastricht University Medical Centre

**Protocol signature sheet**

| **Name** | **Signature** | **Date** |
| --- | --- | --- |
| **Head of Department:**  Prof. Dr. C. Stehouwer  Internal Medicine |  |  |
| **Principal Investigator:**  Prof. Dr. A.A.M. Masclee  Head of Department  Division Gastroenterology/Hepatology |  |  |
|  |  |  |

**Abstract**

**Background:**

Alcohol consumption is a major health problem worldwide. It affects all systems of the body especially the gastrointestinal tract. Acute or chronic alcohol consumption has deleterious effects on the gastrointestinal mucosa vary from increased intestinal permeability, structural changes to sever destruction of the epithelial lining cells. Human data are still limited and most of the studies were performed in chronic alcohol abusers.

We hypothesize that moderate alcohol drinking also may increase small intestinal permeability and contribute to the subsequent disruption of the tight junction complex. This study may provide more insight into the effects of moderate alcohol drinking on the small intestinal permeability.

**Aims of the study:**

The study aims to investigate the effects of moderate ethanol drinking on the intestinal permeability and the tight junction complex. If there is increase in the intestinal permeability, these effects will be investigated at molecular level.

**Study design:**

The study is designed as a placebo-controlled crossover trial.

**Study populations:**

For the study, volunteers who signed the informed consent form, males, between 18 - 45 years old, physically fit, without liver disease, of Caucasian ethnicity and of normal body weight will participate in the study.

**Interventions:**

During this study, alcohol will be administered intraduodenally to induce changes in the intestinal permeability. The exact dose of ethanol will be determined in the explorative study.

**Primary outcome of the study:**

The primary outcome is to assess the small intestinal permeability by means of sugar permeability testing after intraduodenal administration of ethanol.

**Secondary outcomes of the study:**

The secondary outcomes are to assess tight junction’s structure in the biopsy specimens, to examine effects of ethanol and acetaldehyde on mucosal permeability in biopsy specimens in *ex vivo* circumstances using Ussing chamber methodology and to measure serum and mucosal ethanol and acetaldehyde concentrations.

**Risks associated with participation:**

The possible risks in the study are related to the effects of ethanol on the nervous system such nausea, vomiting and euphoria. There are also possible risks related to the intervention with the gastroduodenoscope e.g. the risks of bleeding at the site of the biopsy and perforation of the viscera.

**Introduction**

Alcohol consumption leads to major health problems worldwide^1^. Especially chronic alcohol consumption has health and social consequences and may lead to dependence as well as other medical problems such as acute and chronic liver disease, acute and chronic pancreatitis, diabetes and ischemic heart diseases^1^. The average amount of alcohol consumed and the drinking patterns vary between geographical areas. The mean consumption of alcohol is highest in Europe and Northern America, and lowest in the Eastern Mediterranean^1^. According to estimates of the World Health Organization (WHO), about two billion people worldwide consume alcoholic drinks on a regular basis. In 2000, the global level of alcohol consumption was estimated to be approximately 5.8 liters of pure alcohol per capita per year^1^.

Most people associate moderate alcohol intake with cardiovascular health benefits, but do not consider the fact that it may increase the risk of gastrointestinal malignancies and alcoholic liver disease (ALD)^1^. Animal and in vitro studies have shown that alcohol can increase the intestinal permeability^2, 3^. Subsequently, also Rao *et al*.^4^ reported that chronic alcohol intake increased intestinal permeability, followed by an excessive influx of endotoxin and bacteria and consequently the risk of liver injury , which might be a crucial element in the development of ALD^5^. These findings suggest that alcohol leads to an increased intestinal permeability, thereby contributing to the development of ALD.

Intestinal permeability is an indicator for the intestinal barrier function, which regulates the passage of molecules down a concentration gradient^6^. There are two different routes for permeation, namely the transcellular route, which allows permeation across cells and the paracellular route, which allows permeation between cells. The latter route is regulated by tight junctions (TJs) between the epithelial cells of the intestinal mucosa (Figure 1)^7^. TJs appear as a network of sealing strands and act as a physical intercellular barrier that controls paracellular transport and restricts the movement of lipids and proteins between the apical and basolateral domains of the cellular membrane^4^***.*** TJs are composed of several transmembrane proteins namely, claudins, the integral membrane proteins occludin and junction adhesion molecules (JAMs). Another group of proteins are located at the cytoplasmic side, the zona occludens i.e. ZO-1, ZO-2 and ZO-3 proteins. It has been suggested that, ZO- proteins have a crucial role in junction formation ^8^, they connect between the TJ complex and the actin cytoskeleton^8^.

Opening of the TJs is regulated through a series of signal transduction pathways, resulting in the increased activity of the myosin light chain kinase (MLCK), which phosphorylates myosin and causes contraction of the cytoskeletal components and conformational changes in the associated structures, the tight junctions^9-11^.

**Figure 1: Structure of the tight junction complex.** Van Itallie, Physiology, 2004 ^7^

Different molecules, such as glucose, amino acids^11, 12^, capsaicin^13^ and probably also alcohol^8^ are able to induce opening of the TJs and thereby increase paracellular permeability.

After ingestion of alcoholic drinks, the ethanol contained within can be present throughout the gastrointestinal tract (GIT). About 20% is absorbed in the stomach and about 75% is absorbed in the small intestine, by simple diffusion^14-17^.

A small amount of alcohol is absorbed and metabolized locally in the oral cavity by microbiota as well as in the oesophagus^18^. In case of ingesting a single high dose of ethanol (1.5 g/kg of body weight), equal levels can be measured in the lumen of the ileum and colon compared to the blood, as ethanol reaches the ileum and colon by diffusion from the vascular space^14, 19^.

Once absorbed in the stomach and intestine, ethanol passes into the portal vein to the liver, the main site responsible for metabolizing ethanol. There are also many extra-hepatic sites capable of metabolizing ethanol, namely stomach, lung and colon^19^. In the colon, small amounts of ethanol are metabolized by the colonic bacteria^19^.

In the liver and to a lesser extent in other organs (i.e. Stomach), ethanol is oxidized to acetaldehyde and finally into acetate, which are defined as the hepatic and gastric first-pass metabolism (FPM) , respectively^20^. In the liver, there are three metabolic pathways with different capacities for oxidizing ethanol, namely, alcohol dehydrogenase (ADH), the microsomal ethanol oxidizing system (MEOS) and catalase. All these pathways oxidize ethanol to acetaldehyde^20^.

Alcohol dehydrogenase pathway is the major pathway capable of oxidizing ethanol in the liver^16^. It involves the cytosolic enzyme alcohol dehydrogenase (ADH)^16^. In human, five classes of ADH have been discovered, class I-V^21^. Class I has many isoenzymes with different metabolic capacities present in the liver, stomach and the lung^18, 20^.

The microsomal ethanol oxidizing system (MEOS), which uses the cytochrome P450 system (CYP2E1) enzyme^22^ is predominantly present in the microsomes of the hepatocytes and induced by chronic alcohol consumption and high concentrations of ethanol (*K*_m_ = 8 to 10 mM, compared with 0.2 to 2.0 mM for hepatic ADH)^20^. Comparable to ADH, CYP2E1 oxidizes ethanol and generates acetaldehyde.

The catalase pathway, located in the peroxisomes is capable of oxidizing ethanol in the presence of hydrogen peroxide (H_2_O_2_)^20^, but is considered only a minor pathway of ethanol oxidation^23^.

The end-product of these pathways, acetaldehyde is oxidized to acetate through the action of the enzyme aldehyde dehydrogenase (ALDH), which is localized in mitochondria ( Figure 2 )^14, 20^.

**Catalase pathway**

**ADH pathway**

**MEOS pathway**

**Figure 2: Ethanol metabolism.** Zakhari, S., Alcohol Res Health,2006 ^20^

As described previously, the intestinal barrier is provided by intact tight junction complex. Disruption of the tight junctions leads to an increase in intestinal permeability, which can be caused by several nutritional and injurious agents, including ethanol^4^. Several researchers have investigated effects of ethanol on intestinal permeability, using cell lines or animal models but very few data exist on the human in vivo situation. In vitro and animal data revealed different effects of ethanol, varying from no detectable change in permeability^24^ to an increase in permeability^3^. The exact mechanisms through which ethanol may lead to an increase in intestinal permeability are not fully understood. Ma *et al*.^8^ showed that ethanol in low non-cytotoxic doses can lead to disruption of the Caco-2 cell tight junction barrier, most likely mediated by activation of myosin light chain kinase (MLCK)^8^. In addition, ethanol also stimulates MLCK-activity, but without affecting MLCK-protein levels^8^. The opening of the tight junctions is a functional and reversible process^8^. On the other hand, several investigators^24-27^ concluded that the cytotoxic and carcinogenic metabolite, acetaldehyde can disrupt the tight junctions in Caco-2 cell monolayer. This disruption is caused by induction of tyrosine phosphorylation^24-27^.

Rao *et al*.^24^ compared the effects of both ethanol and acetaldehyde on intestinal permeability in Caco-2 cell lines. They concluded that acetaldehyde but not ethanol did result in an increased permeability, measured by transepithelial electrical resistance (TEER), sodium chloride dilution and the unidirectional mannitol flux^24^.

Reliable data from human studies on the effect of ethanol on intestinal permeability are scarse^5, 28-33^. In most cases, the experiments have been performed after chronic alcohol abuse in alcohol addicts^5, 29, 31^. Robinson and co-workers concluded that ethanol can induce a transient increase in intestinal permeability using polyethylene glycol 400 (PEG-400). This effect resolved within one day in healthy volunteers after a single high dose of alcohol and within four days in chronic alcoholics^33^. Bjarnason *et al*.^28^ used chromium-51 labelled ethylenediamine tetra acetic acid (^51^Cr-EDTA) as a permeability marker and found that chronic alcohol consumption lead to an increase in intestinal permeability in alcoholic patients^28^ . Parlesak *et al* ^32^ investigated whether ethanol consumption may lead to an increase in intestinal permeability to large molecules in patients with chronic alcohol abuse, using PEG with different sizes (M_r_ 400 - M_r_ 10.000). They showed that chronic alcohol consumption did lead to impairment of the intestinal barrier function and increased permeability to small and large molecules i.e., M_r_ 10,000^32^.

In contrast, the group of Hirsch *et al* ^30^ found no alteration in intestinal permeability in both chronic alcoholics and in healthy individuals, tested after three days of abstinence by a lactulose/mannitol ratio test^30^. Keshavarzian and co-workers investigated the effects of acute and chronic ethanol on the permeability of the gastric and small intestinal mucosa in alcoholics and healthy volunteers^5^. They concluded that chronic ethanol reversibly affects the integrity of small intestinal villi without significantly affecting gastrointestinal permeability. In contrast, a single oral dose of ethanol of 0.8g/kg increases gastroduodenal permeability but has no effect on the lactulose or mannitol permeability of the small intestine^5^.

Only two working groups reported on histological findings and concluded that no microscopic alterations of the gastrointestinal mucosa observed after administration of a single high dose of ethanol and chronic alcohol abuse^5, 28^. Millan *et al*. ^31^ investigated the effect of ethanol on the epithelial villi after intragastric alcohol administration. Biopsies were obtained at the peak of the blood alcohol concentrations and showed significant but transient damage to the villi of the upper small intestine^31^. Beside the controversial results of these histological studies, the mechanistic effects of alcohol on the TJs complex in human intestine have not yet been investigated.

In summary, although many in vitro and animal data clearly indicate that ethanol and its metabolite, acetaldehyde can affect intestinal permeability and epithelial integrity, human data are still limited and most of the studies have been performed in chronic alcohol abusers.

Many people of the general population consume low doses of ethanol on a regular basis and subgroup also incidentally consumes high doses. The effect of these drinking patterns on the intestinal permeability and TJs complex has not yet been studied.

The effect of alcohol on the intestinal permeability may have major consequences on health. It’s generally accepted that an increased intestinal permeability in alcoholic subjects lead to translocation of bacterial endotoxins from the gut lumen into the portal vein and then to the liver^14^. In the liver, endotoxins trigger the immune system and activate the resident macrophages, Kupffer cells. Activation of Kupffer cells leads to production of chemokines (IL-8) and proinflammatory cytokines (IL-1, TNFα), resulting in hepatocytes damage, inflammation, fibrosis and finally cirrhosis^34^.

Increased intestinal permeability has been also found in many gastrointestinal disorders such as celiac disease^35^, irritable bowel syndrome^36^, inflammatory bowel diseases^35, 37^.

Studying of these biological events is not only important for understanding the molecular mechanisms but also for the development of therapeutic agents, probably nutritional that are able to restore or prevent epithelial damage and the subsequent liver injury that may occur.

The aim of the present study is to investigate the effects of “moderate ethanol drinking” (2 drinks per day, equivalent to 20 g of ethanol) on the intestinal permeability and the tight junction complex. For that aim, blood and tissue samples from the proximal small intestine will be collected. A multi-sugar test will be applied to measure the intestinal permeability in healthy individuals.

The dose of ethanol will be based on an explorative study to assess the tolerability and the minimal effective dose to induce intestinal permeability changes as well as the correct timing for biopsy. Ethanol will be administered intraduodenally to overcome interindividual differences of oral, esophageal and gastric ADH activity and to avoid the possible dilution of ethanol by gastric secretions.

**Hypothesis and objectives**

**Hypothesis:**

Moderate ethanol intake increases intestinal permeability in the small intestine in healthy individuals and disrupts the tight junction complex.

**Primary outcome:**

- - To assess intestinal permeability by means of sugar permeability testing after intraduodenal administration of ethanol.

**Secondary outcome:**

- - To assess tight junctions structure and proteins in biopsy specimens after intraduodenal administration of ethanol.
  - To examine effects of ethanol and acetaldehyde on mucosal permeability in biopsy specimens in *ex vivo* circumstances using Ussing chamber methodology
  - To assess serum and mucosal ethanol and acetaldehyde concentrations after intraduodenal administration of ethanol.
  - To assess liver functions after ethanol administration i.e. AST, ALT and γGT.

If intestinal permeability is increased:

- To measure plasma endotoxin levels after ethanol administration.
- To assess serum inflammatory markers levels after ethanol administration.

**Aims of the explorative study:**

The aims of the explorative study are:

- To establish the minimal effective dose of ethanol required to induce increased intestinal permeability.
- To assess the safety and tolerability of the dose.
- To determine the peak of blood ethanol concentration.

**Inclusion criteria / exclusion criteria EXPLORATIVE STUDY**

**Inclusion criteria:**

- - 1. Signed informed consent form.
    2. Male gender to avoid the gender-related differences in ethanol metabolism^38^.
    3. Between 18-45 years to avoid age-related changes in ethanol metabolism^39^.
    4. Normal medical history and physical examination.
    5. Normal liver function tests (i.e. ALT, AST, and γGT) according to the reference values for normal ranges of the liver enzymes at the laboratory of clinical chemistry of Maastricht University Medical Center.
    6. Caucasian ethnicity.
    7. BMI=18 – 30 kg/m^2^.

**Exclusion criteria:**

1. History of gastro-intestinal disorders or abdominal surgery.
2. History of alcohol abuse or current excessive alcohol consumption (> 2 alcoholic beverages per day or > 14 alcoholic beverages per week)^40^.
3. Recent or chronic medications that may interact with ethanol metabolism or intestinal permeability i.e., NSAIDs, benzodiazepines and antidepressants.
4. Smoking.

**General considerations:**

- Diet:
  - Continuation of regular dietary habits.
  - Standardized meals three days prior to each study day.
- Dose of ethanol :
  - 20 grams of ethanol. This will be the starting dose, which will be increased gradually (i.e 30 g maximum) if necessary. (See also the explorative study).

**Inclusion criteria / exclusion criteria DEFENTITIVE STUDY:**

**Inclusion criteria:**

1. Signed informed consent form.
2. Male gender to avoid the gender -related differences in ethanol metabolism^38^.
3. Between 18 - 45 years to avoid age-related changes in ethanol metabolism^39^.
4. Normal medical history and physical examination.
5. Normal liver function tests (i.e. ALT, AST, and γGT) according to the reference values for normal ranges of the liver enzymes at the laboratory of clinical chemistry of Maastricht University Medical Center.
6. Caucasian ethnicity.
7. BMI=18 – 30 kg/m^2^.

**Exclusion criteria:**

1. History of gastro-intestinal disorders or abdominal surgery.
2. History of alcohol abuse or current excessive alcohol consumption (> 2 alcoholic beverages per day or > 14 alcoholic beverages per week)^40^.
3. Recent or chronic medications that may interact with ethanol metabolism or intestinal permeability i.e., NSAIDs, benzodiazepines and antidepressants.
4. Smoking.

**General considerations:**

- Diet:
  - Continuation of regular dietary habits.
  - Standardized meals three days prior to each test day
- Dose of alcohol administration chosen:
- Will be based on the results from the explorative study

**Materials and methods**

**Study design**

**The explorative study**

Five male healthy volunteers will be included in the explorative study. Ethanol 20g will be administered as starting dose, using an intraduodenal catheter. The dose will be increased gradually (i.e 30 g maximum) if necessary. Permeability tests will be performed by a multi- sugar tests. (MEC 08-2-070, MEC 04-168).


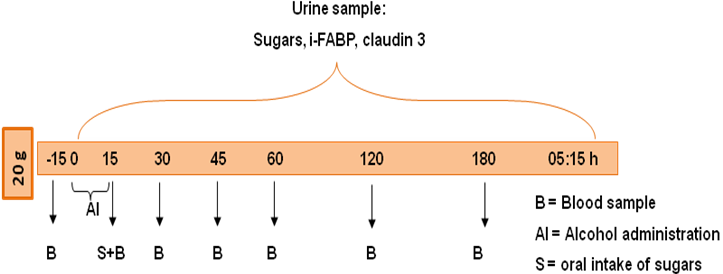


**Figure 3: Schematic diagram showing the design of the explorative study.**

Healthy subjects selected for the explorative study will be instructed to fast overnight from 22:00 PM and will be requested to arrive at the hospital endoscopy unit at 08.00 AM for blood sampling and insertion of the intraduodenal tube. The correct position of the tube will be checked by an X-ray. Once the tube is situated in the duodenum, 20 g of food grade ethanol (EtOH) diluted in tap water to 200 ml (i.e. final concentration of 10 %) will be infused within 15 minutes. Blood samples will be collected at t = -15 min, t = 15 min, t = 30 min, t = 45 min, t = 60 min, t = 120 min and t = 180 min (See figure 3). At t = 15 min, the sugar solution (5 g lactulose and 0.5 g L-rhamnose in 100 ml of tap water) will be ingested orally. Subjects will be requested to remain fasting and only water and tea without sugar will be allowed until the end of the study day. Urine samples will be collected during the first 5 hours.

The time of biopsy sampling for the definitive study will be determined by the peak of blood ethanol concentration.

**The definitive study**

The definitive study will be executed according to a double-blind, placebo-controlled randomized, crossover trial.

The subjects will undergo two study days in a random order, namely ethanol intervention with permeability tests or placebo intervention with permeability tests, with a one week wash out period in between.

**
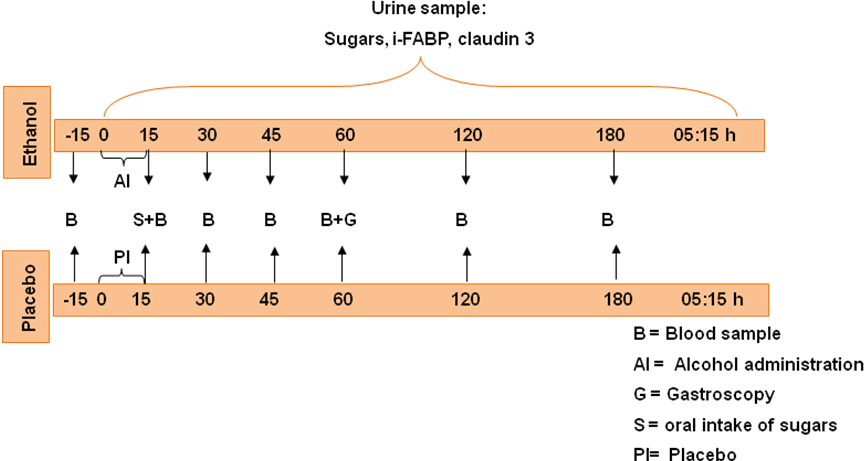
**

**Figure 4: Schematic diagram showing the design of the definitive study.**

Healthy subjects participating in the study will be instructed to fast overnight from 22:00 PM and will be requested to arrive at 8.00 AM at the hospital endoscopy unit for blood sampling and insertion of the intraduodenal tube. The correct position of the tube will be checked by an X-ray. Once the tube is situated in the duodenum, ethanol (dose will be determined from the explorative study) or the placebo (200 ml of tap water) will be infused within 15 minutes. Blood samples will be collected at t = -15 min, t= 15 min, t= 30 min, t= 45 min, t= 60 min, t= 120 min and t= 180 min (See figure 4).

At t= 15 min, the sugar solution (5 g lactulose and 0.5 g L-rhamnose in 100 ml of tap water) will be ingested orally. Subjects will be requested to remain fasting and only water and tea without sugar will be allowed until the end of the study day. Gastroscopy for biopsy sampling will be performed at t= 60 min (will be modified according to the peak of blood ethanol concentration in the explorative study). Urine samples will be collected during the first 5 hours.

**General considerations**

All subjects participating will be requested to refrain from heavy physical exercises. Consumption of alcoholic beverages will be prohibited during one week prior to each study day. In order to standardize food intake among different individuals, three days prior to each day of the study, standard meals will be provided.

The subjects will be fasting overnight from 22:00 h the night before to 08:00 h in the morning each study day. During the study day, subjects will be allowed only to drink water or tea without sugar. Once all tests are performed, a meal will be provided and a hospital bed will be present to rest when necessary. Before the subjects can leave the hospital, ethanol levels will be determined by exhaled breath test. The exhaled breath test will be performed to determine the legal limit of blood alcohol (BAC) concentration 0.05 g/dl (0.05%). Subject will stay at the motility room located at Maastricht University Medical Center (MUMC), under supervision. Transportation home will be provided (taxi).

**Methods**

**Alcohol administration**

Preparations of sugars and ethanol solutions will be performed in a food grade kitchen at Maastricht University (room 3247 UNS 50). The sugars solution will be prepared from 5 g lactulose and 0.5 g L-rhamnose in 100 ml of tap water. The ethanol solution will be prepared from pure food grade ethanol diluted in tap water up to 200 ml (final concentration of 10%). The placebo mixture will consist of 200 ml of tap water.

Because the participants will notice the effects of ethanol as well as the investigator, the solution of ethanol will be prepared and the samples will be recoded by an independent person.

**Blood sampling**

Before and after intraduodenal infusion of ethanol or placebo, blood samples will be collected from an antecubital vein. Each time, 4 mL blood will be collected in 2 K_2_EDTA-tubes to obtain plasma samples, 4 mL will be collected in a SST-tube to obtain serum. The amount of blood will be 84 ml in total. After collection, one K_2_EDTA tube will be centrifuged at 2600 rpm for 20 min at 20°C. The other K_2_EDTA will be centrifuged at 2500 rpm for 15 min at 4°C, the supernatant will be collected and this will be centrifuged again at 4000 rpm for 10 min at 4°C.SST tubes will be kept at 0°C and immediately centrifuged at 2600 rpm for 15 min at 4°C. Serum and plasma will be collected in 1-mL aliquots and stored at -80°C until analysis. Whole blood will be analyzed within 4 hrs and ethanol and acetaldehyde concentrations will be measured immediately.

**Measurements in the blood**

The following parameters will be measured in blood samples, using standard laboratory protocols:

Serum

- Lactulose
- L-rhamnose

Plasma:

- - Circulating cytokines (including IL-6, IL-10, IL-12 and TNF-α).
  - Liver function tests (ALT, AST and γGT).
  - C-reactive protein (CRP), Myeloperoxidase (MPO).
  - Intestinal-type fatty acid binding protein (I-FABP) as a marker of cellular damage.

**Gut permeability test**

Permeability of the small intestine in humans can be assessed non-invasively by measuring the serum and urine excretion ratio of ingested water-soluble, non-degradable test probes. Such a barrier function test is based on a comparison of intestinal permeation of a larger molecule (*e.g.* Lactulose) with that of a smaller molecule (*e.g.* L- rhamnose). For the gut permeability tests, the subjects will have to ingest a sugar drink (100 mL) containing 5 g Lactulose (Centrafarm Services, Etten- Leur) and 0.5 g L-rhamnose (Danisco Sweeteners, Thomson, Illinois, USA) after overnight fasting and collection of urine during the first five hours. The serum and urine ratio of these test molecules represents two distinct routes of permeation (*i.e.* paracellular and transcellular) and provides an estimation of the small intestinal permeability. All sugar probes used in this test are accepted and validated parameters of integrity of the intestinal barrier, and provide an accurate estimation of mucosal damage. Serum will be separated from the blood samples within 30 minutes and stored at -80°C until analysis. Lactulose and L-rhamnose in serum will be determined by fluorescent detection high-pressure liquid chromatography (HPLC) ^41, 42^.

**Duodenal biopsy specimen**

Tissue samples from the horizontal part of the duodenum will be obtained by standard flexible gastroduodenoscope at the endoscopy unit of Maastricht University Medical Center (MUMC) and will be performed by an experienced gastroenterologist. The procedure is widely used in medical practice. Its application in human intervention trials was approved previously by this Medical Ethical Committee (MEC 01-149, MEC 03-196, MEC 05-118). Briefly, in the seated position, an anaesthetizing spray (Xylocaine 2%, Astra Pharmaceutica BV, Zoetermeer, The Netherlands) will be sprayed in the throat to locally anaesthetize the pharynx. A plastic ringlet will be placed in the mouth. The gastroscope will be inserted through the mouth, the oesophagus and the stomach, and into the proximal duodenum, respectively. Eight biopsies of approximately 5 mg of the epithelial tissue will be taken using small (standard) forceps. Upon obtaining the biopsies, the gastroscope will be retracted. The air in the stomach will be aspirated before removing the endoscope. The intestinal mucosa heals quickly from such biopsies.

The biopsy specimens will be used to determine the following:

- Mucosal ethanol and acetaldehyde concentrations.
- Tight junction functionality by
  - Transmission electron microscopy to study the morphological changes of TJs in response to ethanol.
  - Immunofluorescent labelling of perijunctional actin, myosin, ZO-1, myosin light chain kinase, and phosphorylated myosin light chain.
  - *Ex vivo* determination of the effects of ethanol and acetaldehyde on tight junction functionality using Ussing chambers.
- Expression of different genes involved in the regulation of the TJs proteins, ethanol metabolism and the inflammatory cytokines.

**Measurements in the urine**

The following parameters will be measured in urine samples, using standard laboratory protocols:

- Lactulose
- L-rhamnose
- Claudin 3
- I-FABP

**Measurement of alcohol concentration in the exhaled breath**

Blood alcohol concentration will be measured in exhaled breath by the end of each experiment. Alcohol breath testing is an indirect test, but widely used to estimate blood alcohol concentration because of the assumption that an end-exhaled breath sample accurately reflects the alveolar (or deep-lung) air which is in equilibrium with the blood.

**Safety aspects**

**Serious adverse events (SAEs)**

All adverse events reported spontaneously by the subject or observed by the investiga­tor related to ethanol infusion or intraduodenal intubation will be recorded and reported to MEC. The subjects will be closely monitored for ethanol intoxication and therapeutic interventions will be performed accordingly whenever is needed.

The subjects also might get hypoglycemic, therefore, blood glucose levels will be checked hourly. If blood glucose level is < 2.5 mmol/L, intervention with intravenous glucose will be performed.

### Annual safety report

An annual report concerning the safety of the subjects, consisting of a complete safety analysis will be submitted to the MEC.

**Follow-up of adverse events**

All adverse events will be followed until they have abated, or until a stable situation has been reached. Depending on the event, additional tests or medical procedures and/or referral to a medical specialist will be performed accordingly.

**Alcohol administration**

Before starting each experiment, the subjects will be fully examined and routine biochemical analysis including liver function tests will be performed. Preparations of ethanol and sugar solutions will be performed in a food grade kitchen at Maastricht University (room 3247 UNS 50). The solution will be prepared from pure food grade ethanol diluted in tap water up to 200ml (final concentration of 10%).

The subject will not be allowed to leave the hospital before their exhaled breath test levels are within the normal and safe limits.

**Blood sampling**

During blood sampling, the subjects will remain seated in a comfortable chair, with an adjustable back. No side effects are expected when sampling blood in this manner^43^.

**Gut permeability test**

All ingredients of the test beverage (lactulose, L-rhamnose) are used in food industry and are generally regarded as safe. No health risks are associated with the consumption of the test beverage and the subsequent collection of blood. Furthermore, the application of Lactulose and L-rhamnose which will be used has been approved previously by this Medical Ethics Committee (MEC 08-2-070, MEC 04-168).

**Gastroduodenoscopy**

The gastroduodenoscopy, performed by a gastroenterologist, is a standard procedure that takes 10 to 20 minutes. During the procedure 8 biopsies will be taken with a standard biopsy forceps. Diagnostic upper GI endoscopy is a remarkably safe procedure. Although there are no recent high quality prospective studies of complications following diagnostic upper GI endoscopy, one large US study estimated an overall complication rate (including mucosal biopsy) of 0.13% and an associated mortality of 0.004% ^44^. Perforation related to diagnostic upper GI endoscopy is uncommon with an estimated frequency of 0.03 and mortality of 0.001% ^44^.

**Alcohol concentration determination by exhaled breath**

The exhaled breath test is a simple and non invasive technique used for measurement of blood alcohol concentration. It’s also used in roadside studies on the effects of alcohol on driving performance. The subjects will stay at the motility room located at MUMC under supervision and will not be allowed to leave MUMC unless their blood alcohol concentrations are within the legal limit 0.05 g/dl (0.05 % w/v)^45^.

**Ethics**

The study will be conducted according to the principles of the Declaration of Helsinki and in accordance with the Medical Research Involving Human Subjects Act (WMO). Participants will be informed about the study by the principal investigator by both by written information and personal communication.

A time period of one week will be provided to decide whether they would like to participate. All participants will have to sign an informed consent. Participants can at any time make an appeal to the independent doctor, dr. Y. Keulemans (phone 043-3875021) who has agreed to be the independent doctor for this study. Participants will be informed that their decision to participate is totally voluntary and they can withdraw at any time without giving a reason. ~~This will not influence further treatment by their physician.~~ Participants will have the opportunity to be informed about their individual results and the group results at the end of the study.

**Methodological aspects**

**Samples size**

Previous work (F.Troost et al) showed increased small intestinal permeability using indomethacin as stress model. The small intestinal permeability was assessed by the lactulose / rhamnose ratio. The lactulose / rhamnose ratio increased from 0.013±0.009 at the base line to 0.031±0.020 after indomethacin treatment.

According to these data the SD in indomethacin-treated group would be 0.020 and the difference in the mean was 0.018. Considering this difference and using a SD of 0.02, twelve subjects (n=12) are required.

Sample size was determined using the following power calculation program:

[http://biostat.mc.vanderbilt.edu/twiki/bin/view/Main/PowerSampleSize](https://webmail.unimaas.nl/exchweb/bin/redir.asp?URL=http://biostat.mc.vanderbilt.edu/twiki/bin/view/Main/PowerSampleSize) , Version 3.0.

α = 0.05, power = 80%.

**Data analyses**

The dependent variables will be analyzed with the Wilcoxon signed-ranked test, when the distribution of the variables is non-parametric. If the variables are normally distributed a paired t-test will be done.

**Insurance**

Insurance for the participants will be provided in accordance with the legal requirements in the Netherlands (Article 7 WMO and the Measure regarding Compulsory Insurance for Clinical Research in Humans of 23rd June 2003). Maastricht University Medical Centre has got a standard WMO-insurance and liability insurance for research participants.

**Administrative aspects and publication**

**Handling and storage of data**

Data are handled confidentially and coded. The privacy of the participants is guaranteed. All samples and data will be coded in such a way that no personal information about the participants will be available. The key of the code will be kept by the principal investigator, to which only the principal investigator has access. All primary documents and data will be kept for 15 years after the end of the experimental phase of the study for possible inspection. Samples taken from the participants during the study will be kept for 4 years after the end of the experimental phase of the study for additional analysis on epithelial integrity or inflammation.

For future analyses of the study materials, the subjects will not be asked for permission the analyses are within the scope of the present study, Nevertheless, the MEC will be asked for permission to perform these analyses in case the future analyses are not within the scope of the present study.

**Amendments**

All substantial amendments will be notified to the METC that gave a favourable opinion.

**Report progress and end of study**

The investigator will submit a summary of the progress of the trial to the accredited METC once a year. Information will be provided on the date of inclusion of the first subject, numbers of subjects included and numbers of subjects that have completed the trial, serious adverse events/ serious adverse reactions, other problems and amendments.

The investigator will notify the accredited METC of the end of the study within a period of 8 weeks. The end of the study is defined as the last participant’s last visit. In case the study is ended prematurely, the investigator will notify the accredited METC, including the reasons for the premature termination. Within one year after the end of the study, the investigator/sponsor will submit a final report with the results of the study including any publications/abstracts of the study to the accredited METC.

**Publication policy**

All trial results, both positive and negative will be disclosed in agreement with the CCMO statement on publication policy.

Based on the results of this study, at least one publication will be submitted for publication to a peer-reviewed scientific journal. The authorship of the article will be determined in appropriate consultations based on a considerable contribution to the set-up and execution of the study and an active participation in publication. The financial contributor (Top Institute Food and Nutrition) is entitled to examine the manuscript prior to publication and to make comments on it. Within two weeks of the receipt of the draft article Top Institute Food and Nutrition will inform the parties involved whether it wishes for a deferment, to offer Top Institute Food and Nutrition an opportunity to protect its (commercial) interests and rights. Any such deferral will be of a maximum of two months. None of the parties concerned has the right of veto considering publication.

**REFERENCES**

1. WHO Global Status Report on Alcohol 2004. Geneva: Department of Mental Health and Substance Abuse: World Health Organisation, 2004.

2. Draper LR, Gyure LA, Hall JG, Robertson D. Effect of alcohol on the integrity of the intestinal epithelium. Gut 1983;24:399-404.

3. Worthington BS, Meserole L, Syrotuck JA. Effect of daily ethanol ingestion on intestinal permeability to macromolecules. Am J Dig Dis 1978;23:23-32.

4. Rao RK, Seth A, Sheth P. Recent Advances in Alcoholic Liver Disease I. Role of intestinal permeability and endotoxemia in alcoholic liver disease. Am J Physiol Gastrointest Liver Physiol 2004;286:G881-4.

5. Keshavarzian A, Fields JZ, Vaeth J, Holmes EW. The differing effects of acute and chronic alcohol on gastric and intestinal permeability. Am J Gastroenterol 1994;89:2205-11.

6. Travis S, Menzies I. Intestinal permeability: functional assessment and significance. Clin Sci (Lond) 1992;82:471-88.

7. Van Itallie CM, Anderson JM. The molecular physiology of tight junction pores. Physiology (Bethesda) 2004;19:331-8.

8. Ma TY, Nguyen D, Bui V, Nguyen H, Hoa N. Ethanol modulation of intestinal epithelial tight junction barrier. Am J Physiol 1999;276:G965-74.

9. Karczewski J, Groot J. Molecular physiology and pathophysiology of tight junctions III. Tight junction regulation by intracellular messengers: differences in response within and between epithelia. Am J Physiol Gastrointest Liver Physiol 2000;279:G660-5.

10. Shen L, Black ED, Witkowski ED, Lencer WI, Guerriero V, Schneeberger EE, Turner JR. Myosin light chain phosphorylation regulates barrier function by remodeling tight junction structure. J Cell Sci 2006;119:2095-106.

11. Shen L, Turner JR. Role of epithelial cells in initiation and propagation of intestinal inflammation. Eliminating the static: tight junction dynamics exposed. Am J Physiol Gastrointest Liver Physiol 2006;290:G577-82.

12. Pappenheimer JR. On the coupling of membrane digestion with intestinal absorption of sugars and amino acids. Am J Physiol 1993;265:G409-17.

13. Jensen-Jarolim E, Gajdzik L, Haberl I, Kraft D, Scheiner O, Graf J. Hot spices influence permeability of human intestinal epithelial monolayers. J Nutr 1998;128:577-81.

14. Bode C, Bode JC. Effect of alcohol consumption on the gut. Best Pract Res Clin Gastroenterol 2003;17:575-92.

15. Roine R. [First-pass metabolism of alcohol and its clinical significance]. Nord Med 1991;106:325-7.

16. Norberg A, Jones AW, Hahn RG, Gabrielsson JL. Role of variability in explaining ethanol pharmacokinetics: research and forensic applications. Clin Pharmacokinet 2003;42:1-31.

17. Cortot A, Jobin G, Ducrot F, Aymes C, Giraudeaux V, Modigliani R. Gastric emptying and gastrointestinal absorption of alcohol ingested with a meal. Dig Dis Sci 1986;31:343-8.

18. Seitz HK, Poschl G. The role of gastrointestinal factors in alcohol metabolism. Alcohol Alcohol 1997;32:543-9.

19. Salaspuro M. Salaspuro, M. Ann Med 1996;28:195-200.

20. Zakhari S. Overview: how is alcohol metabolized by the body? Alcohol Res Health 2006;29:245-54.

21. Jornvall H, Hoog JO. Nomenclature of alcohol dehydrogenases. Alcohol Alcohol 1995;30:153-61.

22. Agarwal DP. Genetic polymorphisms of alcohol metabolizing enzymes. Pathol Biol (Paris) 2001;49:703-9.

23. Handler JA, Thurman RG. Redox interactions between catalase and alcohol dehydrogenase pathways of ethanol metabolism in the perfused rat liver. J Biol Chem 1990;265:1510-5.

24. Rao RK. Acetaldehyde-induced increase in paracellular permeability in Caco-2 cell monolayer. Alcohol Clin Exp Res 1998;22:1724-30.

25. Atkinson KJ, Rao RK. Role of protein tyrosine phosphorylation in acetaldehyde-induced disruption of epithelial tight junctions. Am J Physiol Gastrointest Liver Physiol 2001;280:G1280-8.

26. Basuroy S, Sheth P, Mansbach CM, Rao RK. Acetaldehyde disrupts tight junctions and adherens junctions in human colonic mucosa: protection by EGF and L-glutamine. Am J Physiol Gastrointest Liver Physiol 2005;289:G367-75.

27. Sheth P, Seth A, Atkinson KJ, Gheyi T, Kale G, Giorgianni F, Desiderio DM, Li C, Naren A, Rao R. Acetaldehyde dissociates the PTP1B-E-cadherin-beta-catenin complex in Caco-2 cell monolayers by a phosphorylation-dependent mechanism. Biochem J 2007;402:291-300.

28. Bjarnason I, Peters TJ, Wise RJ. The leaky gut of alcoholism: possible route of entry for toxic compounds. Lancet 1984;1:179-82.

29. Bode C, Kugler V, Bode JC. Endotoxemia in patients with alcoholic and non-alcoholic cirrhosis and in subjects with no evidence of chronic liver disease following acute alcohol excess. J Hepatol 1987;4:8-14.

30. Hirsch S, Chaves G, Gotteland M, de la Maza P, Petermann M, Barrera P, Bunout D. [Intestinal permeability in alcoholic patients without liver damage]. Rev Med Chil 1997;125:653-8.

31. Millan MS, Morris GP, Beck IT, Henson JT. Villous damage induced by suction biopsy and by acute ethanol intake in normal human small intestine. Dig Dis Sci 1980;25:513-25.

32. Parlesak A, Schafer C, Schutz T, Bode JC, Bode C. Increased intestinal permeability to macromolecules and endotoxemia in patients with chronic alcohol abuse in different stages of alcohol-induced liver disease. J Hepatol 2000;32:742-7.

33. Robinson GM, Orrego H, Israel Y, Devenyi P, Kapur BM. Low-molecular-weight polyethylene glycol as a probe of gastrointestinal permeability after alcohol ingestion. Dig Dis Sci 1981;26:971-7.

34. Tang Y, Forsyth CB, Farhadi A, Rangan J, Jakate S, Shaikh M, Banan A, Fields JZ, Keshavarzian A. Nitric Oxide-Mediated Intestinal Injury Is Required for Alcohol-Induced Gut Leakiness and Liver Damage. Alcohol Clin Exp Res 2009.

35. Teahon K, Somasundaram S, Smith T, Menzies I, Bjarnason I. Assessing the site of increased intestinal permeability in coeliac and inflammatory bowel disease. Gut 1996;38:864-9.

36. Piche T, Barbara G, Aubert P, Bruley des Varannes S, Dainese R, Nano JL, Cremon C, Stanghellini V, De Giorgio R, Galmiche JP, Neunlist M. Impaired intestinal barrier integrity in the colon of patients with irritable bowel syndrome: involvement of soluble mediators. Gut 2009;58:196-201.

37. Oriishi T, Sata M, Toyonaga A, Sasaki E, Tanikawa K. Evaluation of intestinal permeability in patients with inflammatory bowel disease using lactulose and measuring antibodies to lipid A. Gut 1995;36:891-6.

38. Frezza M, di Padova C, Pozzato G, Terpin M, Baraona E, Lieber CS. High blood alcohol levels in women. The role of decreased gastric alcohol dehydrogenase activity and first-pass metabolism. N Engl J Med 1990;322:95-9.

39. Seitz HK, Egerer G, Simanowski UA, Waldherr R, Eckey R, Agarwal DP, Goedde HW, von Wartburg JP. Human gastric alcohol dehydrogenase activity: effect of age, sex, and alcoholism. Gut 1993;34:1433-7.

40. Fan AZ, Russell M, Naimi T, Li Y, Liao Y, Jiles R, Mokdad AH. Patterns of alcohol consumption and the metabolic syndrome. J Clin Endocrinol Metab 2008;93:3833-8.

41. Cox MA, Lewis KO, Cooper BT. Measurement of small intestinal permeability markers, lactulose, and mannitol in serum: results in celiac disease. Dig Dis Sci 1999;44:402-6.

42. Fleming SC, Duncan A, Russell RI, Laker MF. Measurement of sugar probes in serum: an alternative to urine measurement in intestinal permeability testing. Clin Chem 1996;42:445-8.

43. Barrett KE. New ways of thinking about (and teaching about) intestinal epithelial function. Adv Physiol Educ 2008;32:25-34.

44. Silvis SE, Nebel O, Rogers G, Sugawa C, Mandelstam P. Endoscopic complications. Results of the 1974 American Society for Gastrointestinal Endoscopy Survey. JAMA 1976;235:928-30.

45. Smink BE, Ruiter B, Lusthof KJ, Zweipfenning PG. Driving under the influence of alcohol and/or drugs in the Netherlands 1995-1998 in view of the German and Belgian legislation. Forensic Sci Int 2001;120:195-203.
